# Diversified Expression of NG2/CSPG4 Isoforms in Glioblastoma and Human Foetal Brain Identifies Pericyte Subsets

**DOI:** 10.1371/journal.pone.0084883

**Published:** 2013-12-26

**Authors:** Francesco Girolamo, Alice Dallatomasina, Marco Rizzi, Mariella Errede, Thomas Wälchli, Maria Teresa Mucignat, Karl Frei, Luisa Roncali, Roberto Perris, Daniela Virgintino

**Affiliations:** 1 Department of Basic Medical Sciences, Neurosciences and Sensory Organs, University of Bari School of Medicine, Bari, Italy; 2 COMT – Centre for Molecular and Translational Oncology and Department of Biosciences, University of Parma, Parma, Italy; 3 S.O.C. for Experimental Oncology 2, The National Cancer Institute Aviano, CRO-IRCCS, Aviano, Italy; 4 Brain Research Institute, University of Zurich, Department of Health Sciences and Technology, Swiss Federal Institute of Technology (ETH) Zurich, Zurich, Switzerland; 5 Department of Neurosurgery, University Hospital Zurich, Zurich, Switzerland; Biological Research Centre of the Hungarian Academy of Sciences, Hungary

## Abstract

NG2/CSPG4 is a complex surface-associated proteoglycan (PG) recognized to be a widely expressed membrane component of glioblastoma (WHO grade IV) cells and angiogenic pericytes. To determine the precise expression pattern of NG2/CSPG4 on glioblastoma cells and pericytes, we generated a panel of >60 mouse monoclonal antibodies (mAbs) directed against the ectodomain of human NG2/CSPG4, partially characterized the mAbs, and performed a high-resolution distributional mapping of the PG in human foetal, adult and glioblastoma-affected brains. The reactivity pattern initially observed on reference tumour cell lines indicated that the mAbs recognized 48 immunologically distinct NG2/CSPG4 isoforms, and a total of 14 mAbs was found to identify NG2/CSPG4 isoforms in foetal and neoplastic cerebral sections. These were consistently absent in the adult brain, but exhibited a complementary expression pattern in angiogenic vessels of both tumour and foetal tissues. Considering the extreme pleomorphism of tumour areas, and with the aim of subsequently analysing the distributional pattern of the NG2/CSPG4 isoforms on similar histological vessel typologies, a preliminary study was carried out with endothelial cell and pericyte markers, and with selected vascular basement membrane (VBM) components. On both tumour areas characterized by 'glomeruloid' and 'garland vessels', which showed a remarkably similar cellular and molecular organization, and on developing brain vessels, spatially separated, phenotypically diversified pericyte subsets with a polarized expression of key surface components, including NG2/CSPG4, were disclosed. Interestingly, the majority of the immunolocalized NG2/CSPG4 isoforms present in glioblastoma tissue were present in foetal brain, except for one isoform that seemed to be exclusive of tumour cells, being absent in foetal brain. The results highlight an unprecedented, complex pattern of NG2/CSPG4 isoform expression in foetal and neoplastic CNS, discriminating between phenotype-specific and neoplastic versus non-neoplastic variants of the PG, thus opening up vistas for more selective immunotherapeutic targeting of brain tumours.

## Introduction

The most frequent and malignant type of brain tumour, high grade-glioblastoma (WHO grade IV), is an infiltrating and highly vascularized neoplasia that is highly resistant to chemotherapy and radiotherapy and is therefore associated with poor prognosis [1-3]. Neovascularization plays a pivotal role in the propagation and therapeutic refraction of glioblastoma and, accordingly, vessel density is correlated with malignancy and routinely employed as a prognostic indicator [4,5]. Neovessels and their patterning within glioblastoma lesions are morphologically peculiar [6-9], and four principal features of this angiogenic pattern can be distinguished: (1) a high density of vessels at the border of the brain parenchyma suggesting the occurrence of an active sprouting process at the invasive front of the lesion; (2) a similarly active intralesional sprouting generating an enrichment of neovessels within the tumour mass; (3) an endothelial hyperplasia defining garland vessels (a diagnostic criterion for grade IV glioblastoma); and (4) the presence of glomeruloid structures characterized by actively proliferating endothelial cells and pericytes [10].

Numerous prognostic markers have been proposed for gliomas and among these NG2/CSPG4 - a unique transmembrane chondroitin sulphate proteoglycan (CSPG) – has been suggested to be one of the foremost [11-17]. NG2/CSPG4 is characterized by numerous glycosylation sites and three putative glycosaminoglycan (GAG)-attachment sites, making it prone to be expressed as multiple and intricate glycoforms. However, the pattern of expression of such putative isoforms, the modes through which they may be generated, and their precise nature in healthy and neoplastic tissues are still under investigation. In most cases, only one of the three GAG-attachment sites is substituted by a relatively short chondroitin sulphate chain, whereas in several instances (especially in cancer cells) NG2/CSPG4 seems to be intercalated into the plasma membrane as a GAG-free molecule. In its fully glycosylated form NG2/CSPG4 has an apparent molecular weight of >500 kDa and this isoform often coexists with less glycosylated variants running in the range of 250-300 kDa by SDS-PAGE.

The abundance of NG2/CSPG4 in glioblastoma samples is believed to stem from its pronounced expression on early CNS glial progenitors and high levels on pericytes of intra-lesional neovessels [18-25]. This specific expression pattern suggests that NG2/CSPG4 may be an effective therapeutic target for the treatment of cerebral tumours. In fact, siRNA-mediated abrogation of NG2/CSPG4 in human and animal xenograft models of glioblastoma retards tumour growth and suppresses its invasiveness [26] and mouse antibody 9.2.27 in combined immunotherapy with immune cells in GBM-bearing rats may mediate anti-tumour effects [27]. Interestingly, however, although glial precursors are highly dependent upon NG2/CSPG4 and its PDGF-co-receptor activity, experimentally induced gliomagenesis through forced overexpression of PDGF is indistinguishable in wild type and NG2/CSPG4-KO mice [28], suggesting that (at least in the mouse) NG2/CSPG4 is not involved in the inception of glioblastoma through dysregulation of the signalling induced by this proto-oncogene.

A further point of note is that NG2/CSPG4 is normally present on <50% of glioblastoma cells, suggesting that it is associated with specific cell subsets of this tumour. On the other hand, it has not yet been fully established whether NG2/CSPG4 expression coincides with CD133-expressing and non-expressing cancer initiating/stem cells [15,16,29], or whether it identifies alternative, highly malignant cancer cell populations. The possibility that glioblastoma may originate, at least in part, from neoplastic transformation of NG2/CSPG4-expressing glial cells is an attractive hypothesis [19,30], but not fully supported by experimental data [29,31,32].

In light of the potential significance of NG2/CSPG4 in the progression and treatment of glioblastoma, we have generated a wide panel of antibodies against the PG ectodomain and have employed these immunological tools to disclose transitional embryonic-to-adult-to-neoplastic expression patterns of distinct PG isoforms.

## Materials and Methods

### Ethics Statement

Samples of foetal brain were obtained from four post-mortem foetuses derived from spontaneous abortions and received by the Department of Pathological Anatomy, University of Bari School of Medicine. Tissue preparation and storage were as previously described [33], all samples used here were taken post-mortem from the same foetuses. The study was approved by the Ethics Committee of the University of Bari Medical School and complied with the principles stated in the Declaration of Helsinki.

Tissue samples, from glioblastoma patients and controls from temporal lobes after selective amygdalohippocampectomy in patients with chronic pharmaco-resistant mesial temporal lobe epilepsy, were obtained during surgery at the Department of Neurosurgery, University Hospital Zurich. Written informed consent was obtained from patients before study entry. All procedures were conducted in accordance with the Declaration of Helsinki and the study was approved by the Ethics Committee of the Canton Zurich.

Immunizations, blood sampling and euthanizing of animals were performed in compliance with standard protocols approved by the Italian Ministry of Health Committee and by the institutional Bioethics Committee of the National Cancer Institute Aviano, CRO-IRCCS, Aviano, Italy, emanated through the local Animal Care and Use Committee, with the support of the Veterinary Surveillance Unit. The protocols involved all preventive measures to avoid animal distress and suffering. Female Balb/c mice used for monoclonal antibody production were kept in specified pathogen-free conditions, experiments were performed on 4-6-weeks old females bred and housed in the animal facility of the CRO-IRCCS.

### Production of anti-NG2 antibodies

Female Balb/c mice were immunized with the recombinant His-tagged extracellular portion of human NG2/CSPG4 (amino acids 1-2184 of the protein inserted into a pEF6V5his vector) produced in HEK293 cells by repeated intraperitoneal injections of the immunogen solubilized in complete Freund’s adjuvant (Sigma-Aldrich, St Louis, MI, USA). From ELISA screenings of 63 hybridoma clones generated by fusion of NS1 murine myeloma cells with spleen cells from mice that developed an anti-NG2 immune-response, we selected 48 clones for further characterization. Reactivity traits of these clones were initially established by immunohistochemistry, using sections from paraffin-embedded 22-week-old human foetal brain tissues and glioblastoma sections (s*ee below*), and by immunoblotting using the immunogen and a number of human melanoma and soft-tissue sarcoma cell lines (s*ee below*). A number of hybridoma clones that exhibited unique immunostaining and immunoblotting banding patterns were further subcloned, their Igs isotyped using the Pierce^®^ Rapid ELISA Mouse mAb Isotyping kit (ThermoScientifics Inc), and used for the production of ascites fluids in Balb/c or athymic mice.

### Other antibodies

The anti-NG2 polyclonal antiserum D2 was kindly provided by William Stallcup (The Sanford-Burnham Institute for Medical Research, La Jolla, CA, USA). In our previous study we found that in the foetal CNS this antiserum recognizes NG2/CSPG4 isoforms expressed by angiogenic pericytes, but does not react with NG2/CSPG4 of oligodendrocyte precursors [34]. Hybridoma cells producing the anti-NG2 mAb B5/M28 were obtained from ATCC (American Tissue and Cell Collection, ATCC, Rockville, MD, USA). The PE-conjugated anti-NG2/CSPG4 mouse mAb 7.1 was purchased from Beckman-Coulter. The antiserum against β-actin was purchased from Sigma-Aldrich.

### Cell-ELISA

Human cell lines utilized were as follows: A375, M2 and COLO-38, melanoma; SK-UT-1 and SK-LMS-1, leiomyosarcoma, HT1080, fibrosarcoma; 143B, osteosarcoma; all cell lines were from ATCC. Cells were cultured in DMEM (Dulbecco’s modified Eagle’s medium, Life Technologies Inc), low glucose (1.0 g/L), supplemented with Pen/Strep, L- Glutamine and 10% FBS (Foetal Bovine Serum; Life Technologies Inc., Carlsbad, CA, USA). For Cell-ELISA, cells were seeded in 96 well plates at a density of 10,000 cells/well in the above medium. Cells were washed, fixed with 2% paraformaldehyde (PFA) for 30 min, washed and endogenous peroxidase activity blocked by incubation with 3% H_2_O_2_ for 15 min at room temperature. For reducing non-specific Ig binding, cells were further incubated with a blocking buffer (PBS with 2% BSA, 10% sucrose, 0.1% NaN_3_) for 30 min at room temperature. Cells were then incubated overnight at 4°C with the anti-NG2/CSPG4 mAbs, diluted in blocking buffer. The Streptavidin/Biotin/HRP system (Thermo Scientific, TS-125-HR and TM-125-BN) and TMB as a HRP substrate (Sigma-Aldrich) was then employed as recommended by the manufacturer for the detection of antibody binding. All assays were performed at least in triplicate.

### Flow cytometry

Cells were detached from the substrate and collected by incubation in 5 mM EDTA. After extensive washing in PBS, they were re-suspended in PBS at a concentration of 5x10^6^ cells/ml and a volume of 50µl of this cell suspension was incubated for 15 min at room temperature with 1µl of mouse IgG-PE or 20µl of mAb 7.1-PE. Cells were then washed with PBS and re-suspended in 300µl PBS for analysis. All measurements were performed with a FACSCalibur (BD Biosciences, Inc., San Jose, CA, USA) using 10,000 gated events for each sample. A gate was set during acquisition on the forward scatter versus side scatter plot to exclude possible cell debris. The positivity for NG2/CSPG4 was determined by examining the F2 channel at a value of 543 for HT1080 cells used as reference cells.

### Human glioblastoma, healthy and foetal brain samples

Patients underwent conventional contrast-enhanced MRI using a 1.5T MR system (Siemens, Avanto, Philadelphia, PA, USA) with an 8 channels head coil at baseline. Each scan was evaluated for the tumour characteristics (lesion size at greatest diameter in any acquisition plane, location, contrast enhancement, perilesional oedema, and necrosis) by a neuroradiologist of the Institute of Neuropathology, University Hospital Zurich. Tumour localization was the bifrontal/corpus callosum (n=3), right occipito-parietal (n=2), left parietal/corpus callosum (n=3), left post-central/left thalamus (n=1), right fronto-insulo-temporal (n=4), right temporal (n=3); specimens size ranged from 2x3x2cm to 3x4x5.5 cm. Histological diagnosis, on tumour samples from sixteen glioblastoma patients and on parahippocampal control samples from six patients with chronic pharmaco-resistant mesial temporal lobe epilepsy, was performed by routine clinical neuropathology examination at the same institution. Glioma samples were classified according to the WHO criteria and submitted, together with control specimens, to the same histological and IHC procedures as described above for foetal samples.

Samples of foetal brain were collected post-mortem from four 22-week-old human foetuses that did not reveal macroscopic structural abnormalities at autopsy and/or microscopic malformations of the central nervous system after conventional histological analysis with H&E or toluidine blue stainings. The foetal age was estimated on the basis of the crown-rump length and/or pregnancy records (counting from the last menstrual period). The dorso-lateral wall of the telencephalic vesicles (future neocortex) was dissected and fixed in small 0.5 cm thick samples for 2-3 hrs at 4°C by immersion in 2% PFA plus 0.2% glutaraldehyde in a phosphate buffered saline solution (PBS, pH 7.6). Each sample was then washed in PBS and divided into two equal parts, one of which was embedded in paraffin wax and one stored at 4°C in PBS plus 0.02% PFA.

### Immunohistochemistry

Samples of foetal telencephalon, control tissue, and glioblastoma embedded in paraffin were cut into 5-µm serial sections and collected on Vectabond^TM^ treated slides (Vector Laboratories Inc., Burlingame, CA, USA). Sections were rehydrated in blocking buffer-PBS (BB; 1% bovine serum albumin, 2% FCS), treated with 1% H_2_O_2_ in 90% methanol for 20 min at room temperature to quench endogenous peroxidase activity, washed twice in BB, incubated with one of the anti-NG2/CSPG4 monoclonal antibodies (mAbs; 48 original hybridoma clones and 134 subclones for a total of 182 different primary antibodies) overnight at 4°C, and then sequentially incubated with HRP-streptavidin (Vector Laboratories) and the substrate-chromogen 3-amino-9-ethylcarbazole (AEC, Vector Laboratories). Sections were finally counterstained with haematoxylin and coverslipped with Glycergel (Dako Italia, Milan, Italy). For negative controls the primary antibody was omitted or pre-incubated with an excess of recombinant NG2/CSPG4. Staining intensity was assessed blindly on a multi-level score by two independent observers.

Through this initial screening we were able to cluster the entire set of anti-NG2/CSPG4 antibodies into four classes according to their staining pattern. Within each class a prototype antibody was selected, in virtue of its staining reproducibility and intensity, to be applied in the subsequent analyses. Single and multiple immunolabellings were carried out with the following polyclonal antisera (pAbs) and monoclonal antibodies (mAbs), all diluted in BB: mAb anti-CD31, pAb anti-CD105/endoglin, mAb anti-MMP-2, pAb anti-Glut-1, pAb anti-NG2 D2 (generous gift from William B. Stallcup), mAb anti-CD248/endosialin, clone B1/22.4 (generous gift from Prof. Claire M. Isacke, The Breakthrough Breast Cancer Research Centre, London, UK), mAb anti-phospho-PDGFR-β (Tyr751), pAb anti- PDGFR-β, mAb anti-αSMA, pAb anti-collagen type IV, mAb anti-collagen type IV, pAb anti-collagen type VI, pAb anti-CD3, mAb anti-CD45, mAb anti-CD146, mAb anti-O4 (Table S1). Tissue sections were allowed to attach to polylysine slides (Menzel-Glaser, GmbH, Braunschweig, Germany) by drying for 10 min at room temperature (RT), rehydrated with PBS for 5 min at RT, and (for anti-CD105/endoglin) processed by microwave pre-treatment in 0.01 M citrate buffer (pH 6.0) for 15 min at 750 W. They were then incubated with 0.5 % Triton X-100 in PBS for 30 min at RT and with BB 30 min at RT, followed by incubation with single or combined primary antibodies overnight at 4°C and incubation with appropriate fluorophore-conjugated secondary antibodies or appropriate biotinylated secondary antibodies, always diluted in BB and subsequently revealed by fluorophore-conjugated streptavidin (Table S1). After immunolabelling, the sections were fixed in 4 % PFA for 10 min, counterstained with TO-PRO3 diluted 1:10 K in PBS for 5 min at RT (633; Life Technologies, Inc., Gaithersburg, MD, USA) and coverslipped with Vectashield (Vector). The stained sections were examined under the Leica TCS SP5 confocal laser-scanning microscope (Leica Microsystems, Mannheim, Germany) using a sequential scanning procedure. Confocal images were taken at 250 nm intervals through the z-axis of the sections with 40x and 63x oil immersion lenses. z-stacks of serial optical planes (projection images) and single optical planes were analyzed by Leica confocal software (Multicolour Package; Leica Microsystems). The size of immunostained cells observed in the vessel lumen was evaluated with LASAF SP5 software (Leica Microsystems) using a x63 oil immersion lens. The diameter of microvesicles released by endothelial cells (ECs) and the diameter of NG2/CSPG4-reactive pericyte precursor cells (PPCs) was measured on single optical planes from CD31/Coll IV and pAb NG2 D2/mAb 2161D7-labelled sections (n=15), total vascular fields n=60 and n=23, respectively. The results were expressed as mean ± SD.

### SDS-PAGE and Western blotting

Cells were washed with ice-cold PBS and solubilized at 4°C with RIPA lysis buffer (50mM Tris, pH 7.4; 150mM NaCl; 0.5% Na-deoxycholate, 0.1% SDS, 1% Nonidet P-40, 2mM EDTA, Pefabloc^®^ SC 0.8mM, 1 tablet of “complete mini” Roche) for 15 minutes. Lysed cells were centrifuged and the supernatant was aspirated, transferred to a fresh tube on ice, and quantified by the Bradford method. Protein samples were prepared from -80°C frozen sections from 22-week-old human foetal brains and from glioblastoma lesions. For each kind of tissue, replicates of sixteen sections, 20 µm-thick, 80 mm^2^ wide, were scraped on ice using a wet scraper and immersed in the extraction buffer (20 mM Tris-HCl pH 8.8, 2% SDS, 200 mM DTT, 7M Urea, 1 tablet of “complete mini” Roche, 0.8 mM Pefabloc^®^ SC). Lysates were collected in Eppendorf safe-lock tubes in a final volume of 800 μl of extraction buffer and sonicated at the following conditions: 5% output, 5 sec pulse and 30 sec break cooling on ice, repeating 24 times for a total sonication time of 2 min per sample. The material was then heated up to 100°C for 20 min and the extracts clarified for 15 min at 16,000xg at 4°C and quantified by the Bradford method. Samples were solubilized in 5x SDS-PAGE loading buffer (250 mM Tris-HCl pH 6.8, 2.5% SDS, 35% Glycerol, 0.025% (w/v) Bromophenol blue, 125 mM DTT) and resolved on Tris-HCl 5% or pre-cast 4-15% linear gradient gels (Bio-Rad, Richmond, CA), or native PAGE gels (Life Technologies, Inc.). Precision Plus Protein™ Dual Xtra standards (2–250 kDa) and Unstained HiMark Standards (Life Technology Inc.) were employed as molecular markers. Resolved proteins were transferred overnight onto nitrocellulose membranes and the membrane were saturated with 5% dry milk in TBS containing 0.1% Tween-20. Blocked membranes were incubated with the anti-NG2/CSPG4 mAbs (1:2 – 1:10 dilution for supernatants, or 1:150 dilution for ascites fluids) in blocking buffer. Membranes were extensively washed and incubated for 1 hr at room temperature with rabbit anti-mouse antibodies conjugated to HRP (Sigma-Aldrich). Immunolabelled bands were visualized with the ECL Plus Chemiluminescence detection kit (GE Healthcare, Little Chalfont, UK). A polyclonal antiserum against β-actin was employed as a calibrator (diluted 1:400 in TBS-Tween 0,1%), following revealing with the secondary goat anti-rabbit antibodies conjugated to HRP (Sigma-Aldrich).

## Results

### Production of mAbs recognizing distinct isoforms of NG2/CSPG4

To establish the molecular identity and spatial distribution of vascular and tumour-associated NG2/CSPG4 molecules, as well as to highlight putative foetal-adult-neoplastic transitions in the patterns of PG expression, we generated a panel of 63 murine mAbs against the recombinant ectodomain of human NG2/CSPG4, that were characterized through a repertoire of immune-assays and by immunohistochemistry on tissue sections (*see below*). The hybridoma clones were initially screened against a defined, selected panel of tumour cell lines and from this screening we selected 48 clones that exhibited a sufficiently high reactivity against one or more of the cell lines (Table 1). The diverse pattern of immunoreactivity observed through cell-ELISA and complementary flow cytometry (not shown) further confirmed the ability of the hitherto produced panel of antibodies to react with distinct forms of the PG.

**Table 1:** Binding characteristics of anti-NG2/CSPG4 monoclonal antibodies^1^.

**mAb**	**ELISA^2^**	**Cell-ELISA^3^**				**Flow Cytometry**							**Western Blotting^4^**	
	**NG2^rec^**	**A375^5^**	**SKUT1**	**143B**	**M2**	**SKLMS1**	**SKUT1**	**COLO38**	**HT1080**		**143B**	**M2**	**NG2^rec^**	**A375**
B5/M28^6^	n.a.	+++	+++	+++	++	n.a.	n.a.	n.a.	n.a.	n.a.	n.a.	n.a.	240	240,>250
7.1	n.a.	n.a.	n.a.	n.a.	n.a.	++	+++	++++	+	++++	+++	++++	n.a.	n.a.
2161A4	>3.0	+	+++	++	+	-	-	-	-	-	-	-	130-260	250
2161B1	>3.0	++	++	++	-	+++	++++	++	++++	++++	++++	-	-	-
2161C1	1.32	-	-	-	-	+	-	-	n.d.	n.d.	n.d.	n.d.	240,260,270	280
2161D2	1.21	-	-	-	-	-	-	+	-	n.d.	n.d.	n.d.	130-280	270,280
2161D3	2.47	+++	+	++	-	+++	++	-	++	++++	++	-	250	250
2161D4	0.94	++	-	-	-	-	-	++	n.d.	n.d.	n.d.	n.d.	-	-
2161D7	>3.0	+++	++	++	-	++++	++	+	++++	++++	+++	-	100-250	250
2161E1	2.50	++	+	+	-	+++	+++	++++	++++	++++	+++	-	260	260
2161F8	0.80	-	-	-	-	-	-	++	n.d.	n.d.	n.d.	n.d.	>250	>250
2161F9	0.94	++	++	++	-	++	++	++++	++++	++++	++	-	>200	250,280
2161G7	>3.0	-	-	n.d.	-	+++	-	++++	n.d.	n.d.	n.d.	n.d.	-	-
2161G8	>3.0	-	-	n.d.	-	+	-	++++	n.d.	n.d.	n.d.	n.d.	-	>250
2161G11	1.34	-	-	n.d.	-	-	-	-	n.d.	n.d.	n.d.	n.d.	>250	>250
2161H2	>3.0	++	++	++	-	-	-	++++	++	++	-	-	-	-
2161H3	>3.0	+++	+++	++	-	++	-	-	n.d.	n.d.	n.d.	n.d.	195	250
2161H8	>3.0	++	++	++	-	++++	+++	+	++++	++++	+++	-	-	-
2163A1	>3.0	++	-	-	+	+++	-	++	n.d.	n.d.	n.d.	n.d.	-	-
2163C3	>3.0	+++	++	+	-	++	+	++++	++	++++	-	-	>250	>250
2163D7	1.27	++	++	++	-	++	+++	++	+++	++++	+	-	100-270	130,260,280
2163F4	>3.0	++	++	-	-	-	-	++	+	-	-	-	130-280	>250
2163H4	>3.0	+++	++	++	-	++++	+++	++++	++++	++++	++++	-	-	-
2164A2	1.68	+++	++	++	-	++++	+++	++++	++++	++++	++++	-	-	-
2164A7	>3.0	++	+	++	-	-	+	+	n.d.	n.d.	n.d.	n.d.	100-260	120-280
2164B6	>3.0	+++	++	-	-	++++	+++	+++	++++	++++	+++	-	>130	250
2164C3	>3.0	++	+	-	-	++++	++	++++	++++	++++	++++	-	>150	150,200,>250
2164G7	>3.0	++	++	++	-	++++	+++	++++	++++	++++	++	-	-	-
2164H5	1.99	-	+++	+	-	-	-	-	++++	n.d.	n.d.	n.d.	>250	>250
2164H9	>3.0	-	+	-	-	-	-	+	n.d.	n.d.	n.d.	n.d.	-	-
2166G4	>3.0	++	++	+++	-	-	-	++	++	+++	-	-	250	50-250
2166G2	0.53	n.d.	-	-	-	-	-	-	n.d.	n.d.	n.d.	n.d.	-	-
2172A2	2.01	-	+	++	-	++	++	-	+++	++++	++	-	130-270	130-270
2172B5	2.28	+++	+	++	-	-	-	-	n.d.	n.d.	n.d.	n.d.	>250	>250
2172B12	>3.0	-	+	-	-	+++	++	+	++++	++++	++	-	100-280	280
2172C2	>3.0	-	++	++	-	++++	++++	++	++++	++++	++++	-	>250	>250
2172C3	1.9	-	-	-	-	+	++	++	++	++	+	-	95-250	>250
2172C10	2.05	-	-	++	-	+++	+	++++	+++	++++	++	-	-	-
2172C12	>3.0	++	++	+++	-	+	-	+	n.d.	n.d.	n.d.	-	-	-
2172D6	>3.0	++	+	+	-	++	++	+++	++++	++++	++	-	130-280	240-280
2172D12	2.82	-	-	+	-	-	-	-	-	-	-	-	>250	>250
2172E8	>3.0	++	-	+	-	+	-	++	n.d.	n.d.	n.d.	n.d.	240,270,280	270,280
2172E9	>3.0	++	-	+	-	-	-	+	n.d.	n.d.	n.d.	n.d.	>250	>250
2172F4	2.35	++	-	+	-	-	-	+	n.d.	n.d.	n.d.	n.d.	250	>250
2172F11	1.18	-	-	-	-	-	-	+++	-	-	-	-	>250	>250
2172G3	1.66	-	-	-	-	++++	++	++++	++++	++++	++	-	-	-
2172G4	1.39	-	-	-	-	+++	++	-	++++	++++	++	-	-	-
2172G6	1.26	-	-	-	-	++	+++	-	++++	++++	++	-	-	-
2172H6	2.83	-	+	-	-	+++	++++	++++	++++	++++	+++	-	-	-
2172H12	2.50	++	-	+++	-	-	-	+	n.d.	n.d.	n.d.	n.d.	250	>250

^1^ Detailed analyses of the binding properties of the antibodies were performed on a selection of 49 of the 63 hybridoma clones originally produced. ^2^ Performed as a solid-phase binding assays with HRP-based colorimetric detection on the recombinant NG2/CSPG4 extracellular fragment used as immunogen. ^3^ Performed as a solid-phase binding assay with HRP-based colorimetric detection on PFA fixed cells. ^4^ Performed on material resolved by SDS-PAGE (4-15% gradient gels) under reducing conditions. ^5^ Human cell lines were as follows: A375, M2 and COLO-38, melanoma; SK-UT-1 and SK-LMS-1, leiomyosarcoma; HT1080, fibrosarcoma; 143B, osteosarcoma. ^6^ MAbs B5/M28 and PE-conjugated 7.1 were used as reference antibodies for Western blotting and flow cytometry, respectively. Semi-quantitative positivity scoring for cell-ELISA and flow cytometry was as follows: “-”, 0-15%; “+”, 15-25%; “++”, 25-50%; “+++”, 50-75%; “++++”, 75-100%. Abbreviations: n.a., not applicable; n.d., not determined.

Most mAbs were found to detect the widely observed NG2/CSPG4 doublet migrating at *M*_*r*_ 230-260 kDa and previously proposed to correspond to two of the most frequently occurring PG variants encompassing the entire ectodomain, but differing as regards the presence or absence of the N-terminal Ca^2+^-binding module (Table 1). These NG2/CSPG4 variants also appeared to be weakly glycosylated and virtually completely non-glycanated. Whether the seemingly GAG-free NG2/CSPG4 variants recognized by the antibodies corresponded to subsets of the PG molecules expressed on the surfaces of the examined cell lines, or whether they corresponded to the representative NG2/CSPG4 molecules of these cells, remains to be established. Antibodies 2161F9 and 2161D7 additionally detected a series of proteolytic fragments of NG2/CSPG4 presumed to have been generated by endogenous proteases of HEK293 cells in which the ectodomain fragment was expressed as a recombinant protein. Typical endogenous proteolytic fragments had an apparent *M*_*r*_ of 100-150 kDa (Table 1). Antibody 2161D3 preferentially reacted with a somewhat smaller variant of NG2/CSPG4 running at 220-230 kDa, as well as a substantially more glycosylated isoform in A375 melanoma cells (Table 1).

### Identification of distinct isoforms of NG2/CSPG4 on glioblastoma and foetal brain sections

In the foetal human cortex, lodging active angiogenic processes, sprouting vessels and newly formed, still poorly stabilized vascular structures are rich in NG2/CSPG4-expressing pericytes, which we have previously shown to be primary actors in the formation of these nascent vessels [34]. By contrast, NG2/CSPG4 expression is downregulated in pericytes associated with quiescent vessels [14], and in adult human brain we found that NG2/CSPG4 is virtually absent in pericytes of stable microvessels [34] (see also Figure S1A-F). This would indicate that glioblastoma neovessels, known to be composed of NG2/CSPG4-expressing pericyte subsets, are more similar to the developing than to the adult vasculature.

Starting from these basic observations, comparative immunolabellings were performed on paraffin sections of both human foetal brain and glioblastoma with the 48 clones resulting from the immune-assays screening. These 'in situ' experiments identified a total of 14 clones, of the 48 tested, that detected differently distributed cell variants of the NG2/CSPG4 PG as well as the presence of cell surface-shed NG2/CSPG4 fragments that remained entrapped in the ECM (Table 2). Four classes of mAbs were identified according to the qualitative and quantitative differences observed in revealing the corresponding NG2/CSPG4 isoforms (Table 2). For each class, a prototype clone (i.e. mAbs 2164H5, 2161F9, 2161D7, and 2166G4; Table 2 and Figure S2), characterized by its distinctive and intense IHC signal, was designed to represent its distributional class in the subsequent confocal microscopy analysis.

**Table 2:** Anti-human NG2/CSPG4 proteoglycan clones selected by IHC on paraffin-embedded samples of glioblastoma and foetal brain and listed according to their distributional staining pattern^1^.

**mAb**	**Glioblastoma**				**Foetal brain**		
	**ECM**	**PCs**	**PPCs**	**TCs**	**ECM**	**PCs**	**OPCs**
**2164H5^2^**	++++	+++	-	-	+++	+++	-
2172D12	+++	++	-	-	++	++	-
2172F11	+++	++	-	-	++	++	-
2161D4	++	++	-	-	++	++	-
**2161F9**	-	++++	+++	-	-	++++	-
**2161D7**	-	++++	-	-	-	++++	+++
2161A4	-	-	-	++	-	-	-
2164G7	-	+++	-	-	-	++	+
**2166G4**	-	-	-	++++	-	-	-
2172B12	-	-	-	++	-	-	-
2172C3	-	-	-	++	-	-	-
2172C12	-	-	-	++	-	-	-
2172D6	-	-	-	+	-	-	-
2172H6	-	-	-	+	-	-	-

^1^ Detailed analyses of the IHC properties of the antibodies were performed on a selection of 48 of the 63 hybridoma clones originally produced.^2^ In 'bold' the prototype hybridoma clone for each identified isoform class.. Semi-quantitative scoring of IHC reactivity: “-“, very weak or absent; “+”, weak; “++”, intermediate ; “+++”, strong; “++++”, very strong. Abbreviations: ECM, extracellular matrix; PCs, pericytes; PPCs, pericyte precursor cells; TCs, tumoral cells; OPCs, oligodendrocyte precursor cells.

### Immunohistochemical characterization of glioblastoma neovessels

**Figure 1:**
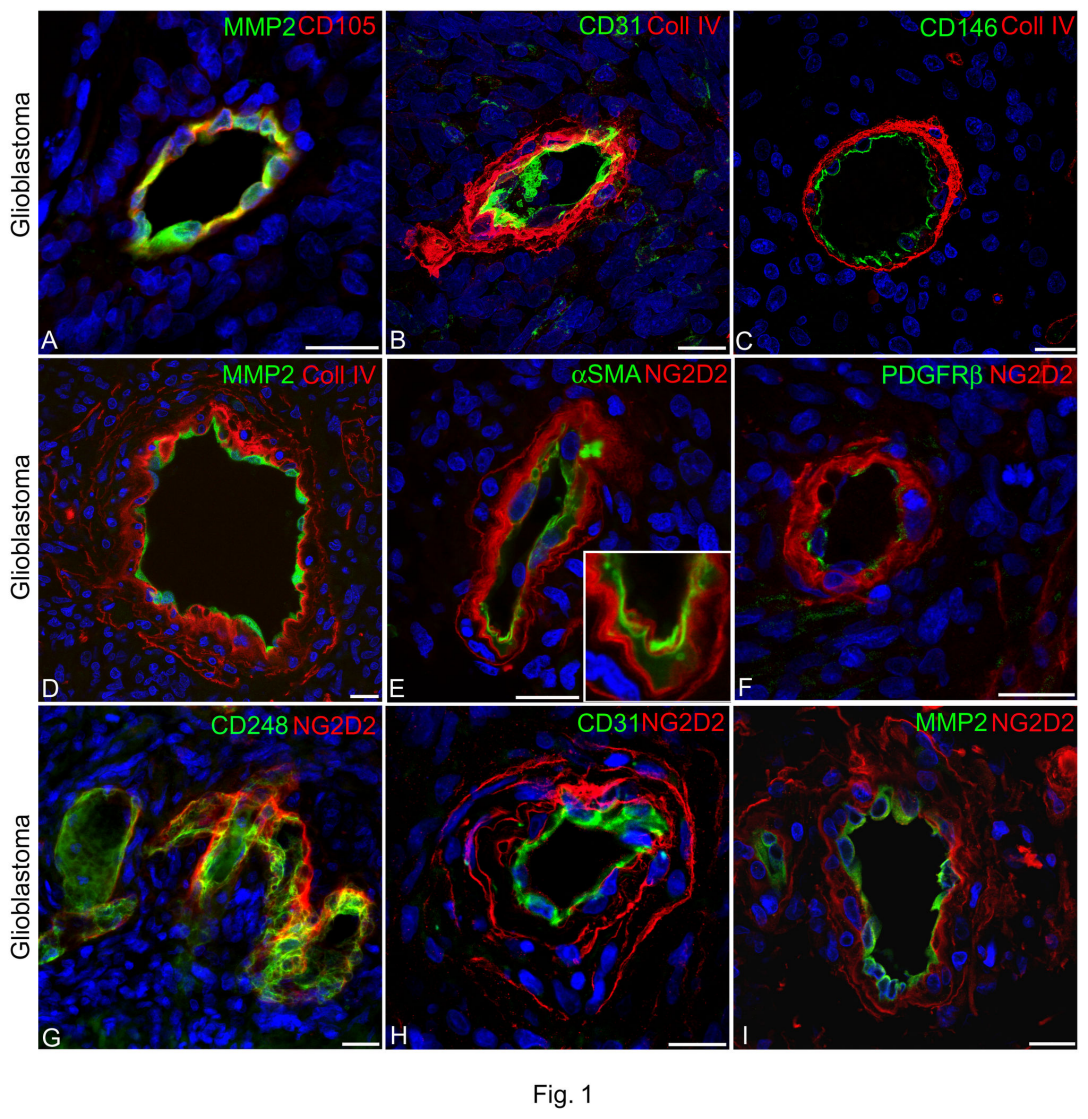
Spatial organization of ECs and pericytes within the glioblastoma neovessel wall in relation to the multilaminar VBM. (**A**) Colocalization of MMP2 and CD105 on tumoral ECs; (**B**-**D**) relative localization of CD31-, CD146-, and MMP2-reactive ECs in tumour vessels characterized by a thick, multilaminar Coll IV VBM. (**E**-**G**) The multilayered arrangement of NG2/CSPG4 D2-expressing pericytes and the relative innermost localization of αSMA (**E**), PDGFRβ (**F**), and endosialin/CD248 (**G**) immunolabelled pericytes. (**H**, **I**) Perivascular distribution of cell surface-shed fragments of the PG NG2; note in (**C**) restricted Mel-CAM/CD146 reactivity on the luminal EC plasma membranes, in (E, inset) the alternate αSMA cytoplasmic reactivity and cell and membrane-associated NG2 D2 labelling, and in (**F**) the activated form of PDGFRβ revealed on the adluminal pericyte plasma membrane. Nuclear counterstaining TO-PRO3. Bars 20 µm.

Heterogeneity in tumour microvascular architecture includes a variable expression of EC (endothelial cell) and pericyte markers and reflects the origin, density, structure, and tissue distribution of the newly formed vessels. For this reason, we firstly applied a panel of vessel markers that could define in more detail (i) the structural and compositional properties of intra-lesional vessels of glioblastoma, (ii) the tumoral areas characterized by a precise vessel typology denoted 'glomeruloid bodies' and 'garland vessels', both described as forms of endothelial and pericyte proliferation [10,35,36], and (iii) the spatial organization of ECs and pericytes also in relation to their surrounding vascular basement membrane (VBM). Preliminary, peripheral tumour areas, as well as areas where necrotic, pseudopalisading and/or haemorrhagic aspects prevailed, were not included in the study (Figure S3). Reference EC markers were the ubiquitous junction-associated Ig-like protein PECAM-1/CD31, the enzyme MMP2, the transmembrane receptor endoglin/CD105, and the integral membrane glycoprotein Mel-CAM/CD146, all of which are highly expressed in angiogenically active ECs and in metastatic processes [37-41] (Figure 1A-D, H, I). Mature and activated pericytes were immunolocalized by αSMA, PDGFRβ, endosialin/CD248 [34,42], and NG2/CSPG4 D2 (Figure 1E-I; Figure S4A-F). VBM was delineated by collagen type IV (Coll IV) (Figure 1B-D). The typical multilaminar arrangement of the tumour VBM, that is a hallmark of the "garland vessels" phenotype [10,35,36,43], was revealed on sections immunolabelled with anti-Coll IV and with NG2/CSPG4 D2 antiserum, which also recognized fragmented PG, possibly released by cell surface proteolysis (Figure 1D, E, H, I; Figure S4A, C, D, F). EC markers showed monolayers of activated MMP2/CD105-reactive ECs (Figure 1A, D, I), while CAM/CD146 characteristically decorated the luminal EC membrane (Figure 1C) and CD31 revealed microvesicles-like structures showing a diameter ranging from 1.57 to 3.44 µm, which appeared to be detached from the luminal EC membrane (Figure 1B and Movie S1). Pericytes were revealed to be organized in multistrata and the detailed immunolocalization of NG2/CSPG4 D2 together with αSMA, PDGFRβ, and CD248 discriminated between two spatially separated populations: one directly facing the EC lining (αSMA-, phospho-PDGFRβ, CD248-expressing pericytes) and one, more externally located, formed by NG2/CSPG4 D2-reactive pericytes (Figure 1E-G; Figure S4A-F; Movie S2). Unlike NG2/CSPG4, that was equally distributed in pericytes, the phosphorylated form of PDGFRβ showed a distinct subcellular polarization with a preferential accumulation at the endothelial-pericyte interface (Figure 1F and Figure S4A-C).

### Differential expression of NG2/CSPG4 isoforms in glioblastoma lesions and foetal brain unveils pericyte subsets

**Figure 2:**
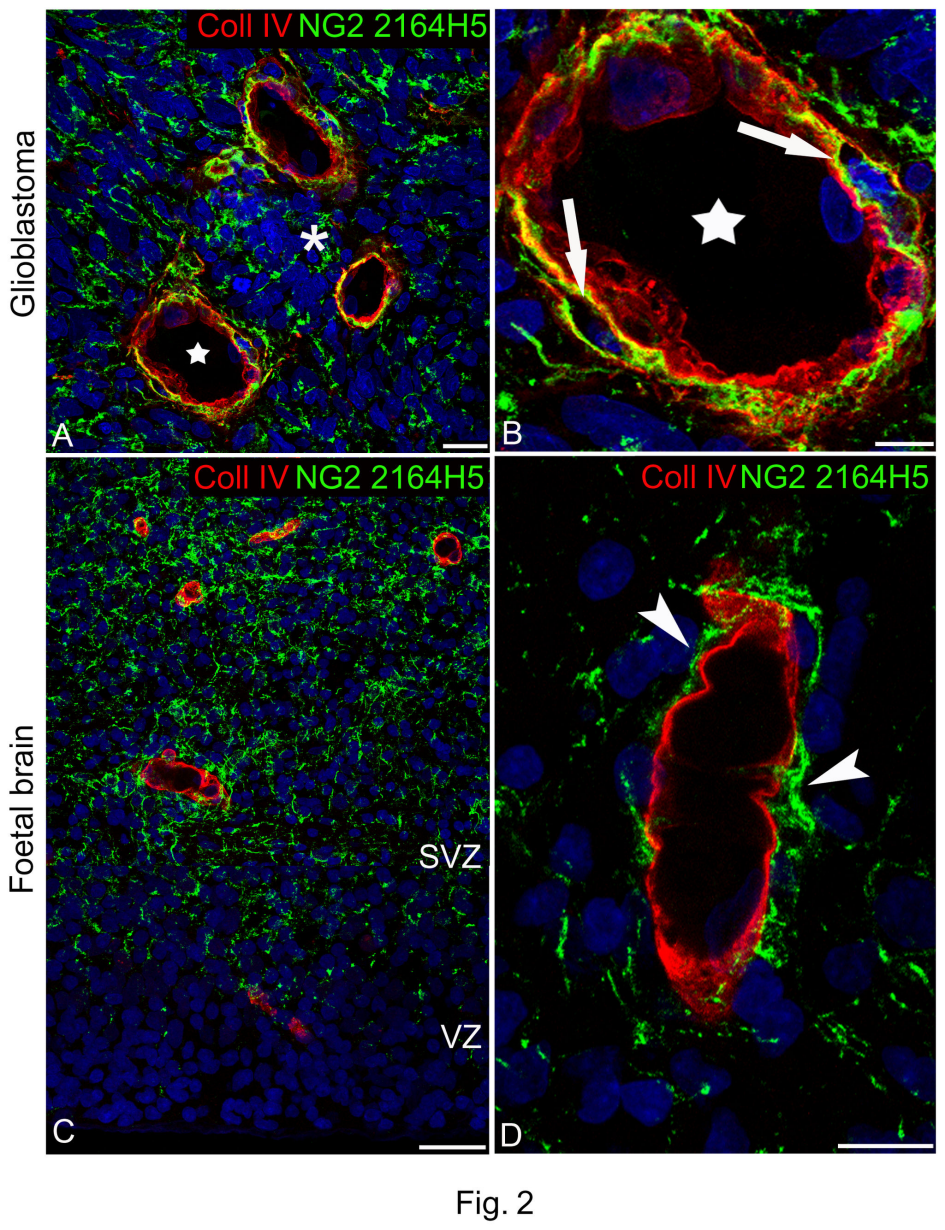
Distribution of NG2/CSPG4 isoforms recognized by mAb 2164H5 in glioblastoma and foetal brain. (**A**) 2164H5/Coll IV double immunolabelling shows 2164H5-reactive NG2/CSPG4 isoforms close to pericytes fully embedded in the VBM; proteolytic fragments of these isoforms appear dispersed through the stromal ECM (asterisk) and decorate glioblastoma cells. (**B**) High magnification view of the neovessel marked with a star in (**A**) in which this specific pericyte subset preferentially localizes in a more abluminal position with respect to the Coll IV-containing VBM, and resides in mural 'pericyte lacunae' (arrows). (**C**, **D**) The distribution of NG2/CSPG4 isoforms recognized by mAb 2164H5 in foetal brain closely resembles that seen in glioblastoma neovessels; notably (**C**), NG2/CSPG4 fragments form a concentration gradient which increases from the ventricular zone (VZ) through the subventricular zone (SVZ) and close to the vessel wall (D, *arrowheads*). Nuclear counterstaining TO-PRO3. Bars A, C 25 µm; B, D 10 µm.

In the glioblastoma neovessels, mAb 2164H5 identified a pericyte subset that was juxtaposed to the Coll IV-containing VBM, but also recognized surface-released fragments of the PG that crusted the outer vessel surface and could be discerned occupying intercellular spaces of the lesion (Figures 2A, B). This suggested that the epitope recognized by mAb 2164H5 in NG2/CSPG4 was retained in the proteolytic fragments of its ectodomain. An analogous distribution pattern of this NG2/CSPG4 isoform was observed in the subventricular zone of foetal brain, where cell surface-shed fragments of the PG were observed in the ECM and around the vessel wall (Figure 2C, D). Interestingly, in the telencephalic ECM, released 2164H5-reactive NG2/CSPG4 fragments seemed to be excluded from the ventricular zone and to be progressively increased through the subventricular zone to the cortical plate (Figure 2C).

**Figure 3:**
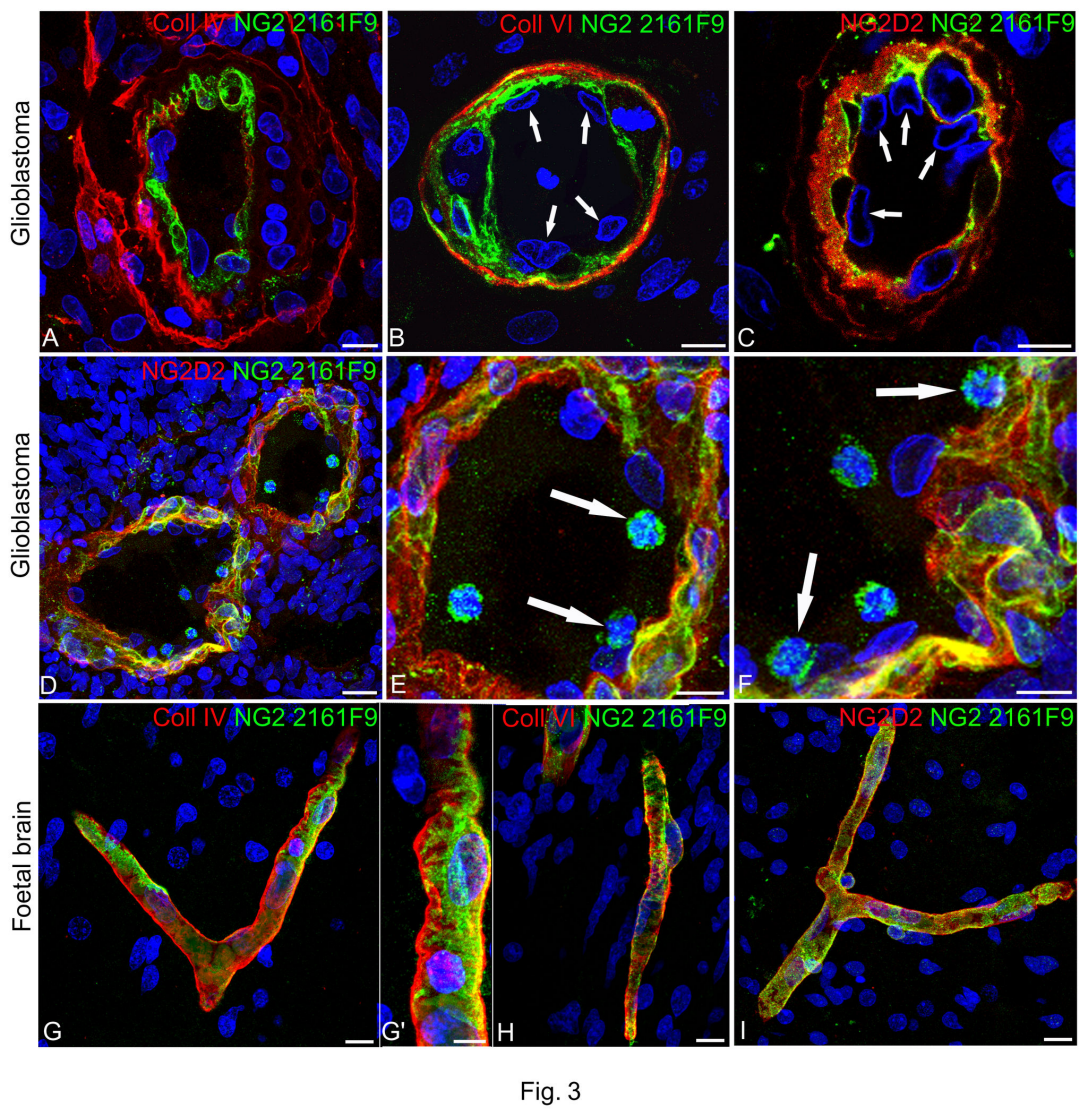
Expression of 2161F9-reactive NG2/CSPG4 isoforms by pericyte subsets. (**A**-**C**) Double immunolabellings of glioblastoma lesions with mAb 2161F9 and antibodies to Coll IV, Coll VI, or the NG2 D2 pAb, specifically show that the pericyte subpopulation expressing these isoforms is organized in the innermost pericyte layers of both 'garland' vessels (**A**) and smaller neovessels characterized by the typical proliferative appearance of EC whose nuclei bulge into the vessel lumen (B, C; *arrows*). (**D**-**F**) Capturing of pericyte precursor-like cells 2161F9-reactive NG2/CSPG4 isoforms within the vessel lumen and adherent to the vessel wall (arrows in E and F, enlargements of D). (**G**-**I**) A pericyte subset expressing the NG2/CSPG4 isoform recognized by mAb 2161F9 is also seen in foetal brain, where its specificity is confirmed, allowing a clear-cut view of the pericyte cell body and processes (high magnification of G in **G**'). Nuclear counterstaining TO-PRO3. Bars A-C 10 µm; D 20 µm; E-G, H, I 10 µm; G' 5 µm.

Double immunolabellings performed with mAb 2161F9 and antibodies to Coll IV and Coll VI (Figure 3A, B) or the pAb NG2/CSPG4 D2 (Figure 3C-F) revealed that in glioblastoma tissues these specific isoforms accumulated at the endothelium/pericyte interface and thereby, as compared with NG2/CSPG4 D2, immunolocalized the innermost pericyte subset (Figure 3C). At this location, the NG2/CSPG4-expressing pericyte subset contacted the Coll IV stratified VBM, whereas Coll VI was organized in a monolayer associated with the pericyte basal side. These pericyte subpopulations showed a different frequency and spatial arrangement in neovascular beds of glioblastoma and the microvessels of foetal brain (Figures 3G-I). Whereas the pericyte population marked by expression of 2161F9-reactive isoforms structured the entire luminal surfaces of glioblastoma vessel walls, in partial continuity with the Coll IV-containing stratified VBM and in a contiguous manner with the surrounding Coll VI (Figures 3A, B), the pericyte phenotype revealed by this isoform in foetal brain corresponds to single, often isolated pericytes, which were clearly seen to embrace the microvessel wall with short processes and partially cover the Coll IV/Coll VI/NG2/CSPG4 D2-containing VBM (Figures 3G-I). Clone 2161F9 in double immunolabelling with NG2/CSPG4 D2 pAb disclosed, together with the described subset of pericytes, a number of intraluminal cells having an average size of ~9µm (8.99µm ± 0.43µm) (Figure 3D-F and Figure S5A-C, A'-C'). Since these cells were found to be negative for CD3, CD31, CD45, CD146 and CD105, they were initially defined as NG2-reactive precursor-like cells and subsequently definitely identified, thanks to double labelling with mAb 2161F9 and the pericyte marker PDGFRβ as pericyte progenitor cells (PPCs) (Figure S5D-F) [44].

**Figure 4:**
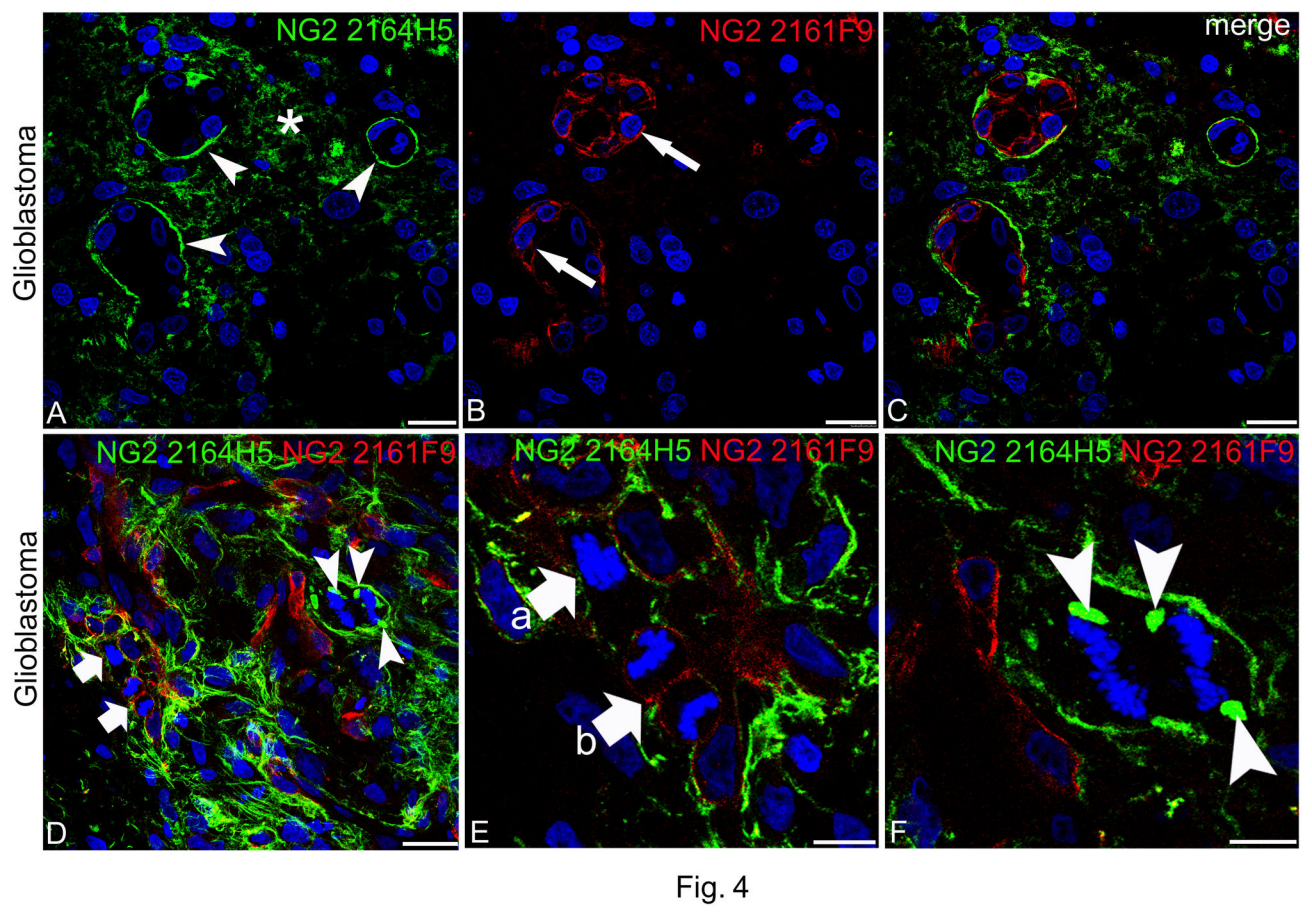
Comparative distribution patterns of two different NG2/CSPG4 isoforms revealed in glioblastoma lesions by double labelling with mAbs 2164H5 and 2161F9. (**A**-**C**) mAb 2164H5 (**A**) primarily discloses cell surface-released fragments of NG2/CSPG4 included in the VBM (arrowheads) and present in the intercellular spaces (asterisk), whereas mAb 2161F9 (**B**) defines the pericyte layer (a*rrows*); on the merged image (**C**), the diverse NG2/CSPG4 expression profiles are clearly recognizable. (**D**) In a tumour area rich in ECM, clone 2161F9 reveals actively dividing pericytes (arrows), shown in detail in (**E**) (arrow a, prophase and *arrow* b, telophase), and two tumour cells, enlarged in (**F**), in advanced anaphase of mitosis (arrowheads); note in (**F**) NG2/CSPG4 aggregates next to the dividing chromosomes (arrowheads). Nuclear counterstaining TO-PRO3. Bars A-C, 20 µm; D, 25 µm; E, F 10 µm.

Evidence of clone selectivity in recognizing different NG2/CSPG4 isoforms was obtained by double immunolabelling with 2164H5 and 2161F9. On these sections, although a close vicinity was noted between the isoforms, the respective signals did not coincide and partially overlapped only on the vessel wall: mAb 2161F9 was restricted to the pericyte subset, whereas mAb 2164H5 reactivity appeared to be more directly related to the VBM and to shed fragments of the PG present in the tumour intercellular spaces (Figure 4A-F). Interestingly, in areas of the lesion where aspects of vascular and tumoral cell proliferation abounded, dividing pericytes still distinctly labelled by clone 2161F9 were seen, together with proliferating tumour cells that, during mitosis, accumulate 2164H5-reactive NG2/CSPG4 within the cytoplasm and close to the dividing chromosome (Figure 4D-F).

**Figure 5:**
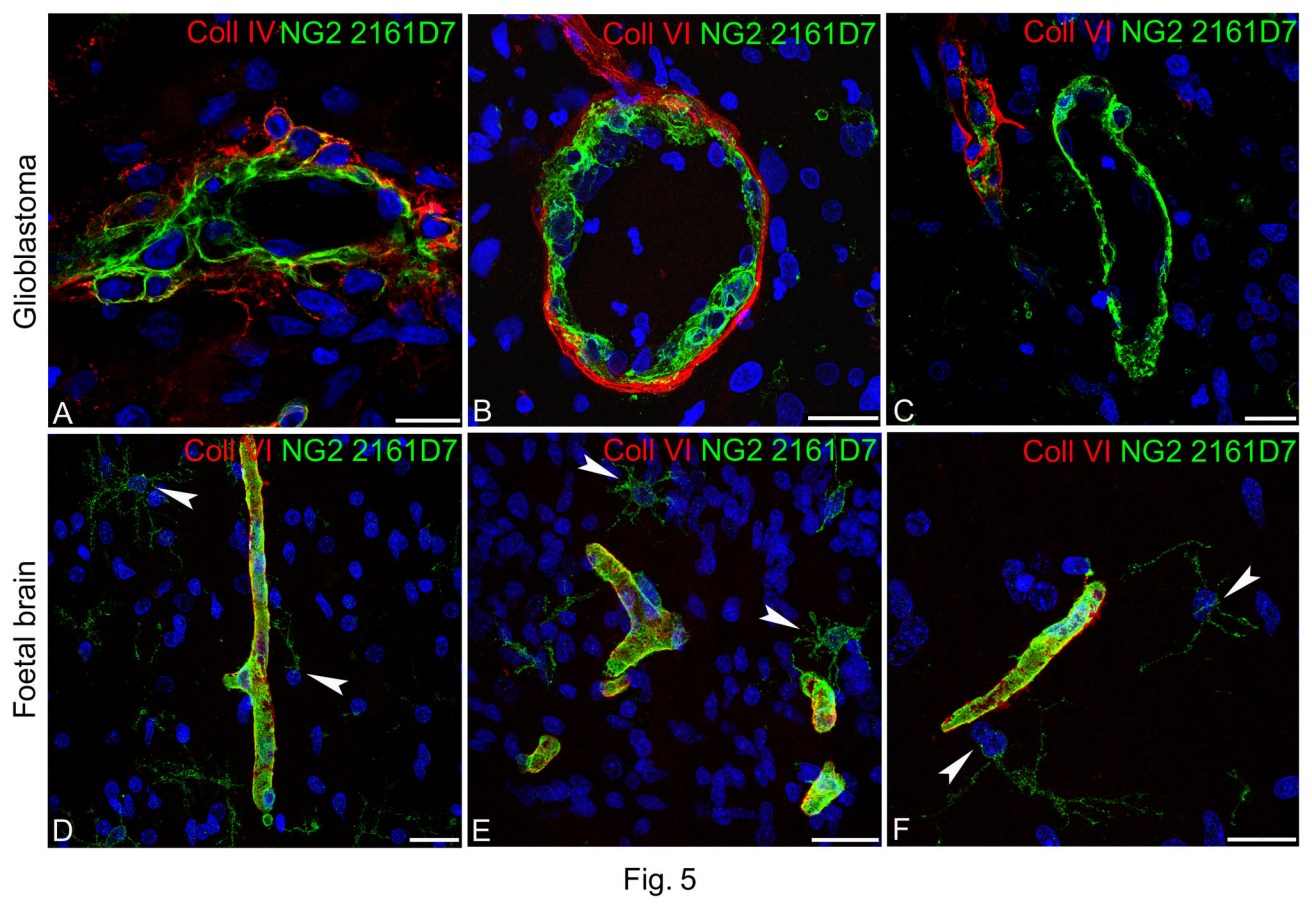
Expression of NG2/CSPG4 isoforms recognized by mAb 2161D7 in glioblastoma and foetal brain. (**A**) NG2/CSPG4 isoforms detected by mAb 2161D7 appear ubiquitously distributed through the pericyte layers, decorating the luminal side of glioblastoma neovessels and co-localizing with the outermost VBM Coll IV layers. (**B**) 2161D7-reactive multilayered pericytes surrounded by Coll VI VBM. (**C**) A 'naked' tumour vessel with a thin layer of pericytes without a detectable Coll VI VBM. (**D**-**F**) Identical double stainings of foetal brain to those performed on glioblastoma sections confirm an analogous spatial arrangement of the pericyte population marked by mAb 2161D7-reactive NG2/CSPG4 isoforms, and additionally highlight the expression of such isoforms on typical foetal OPCs (arrowheads). Nuclear counterstaining TO-PRO3. Bars A, C, F 20 µm; B, D, E 25 µm.

On glioblastoma tissue, prototype mAb 2161D7, and clone 2164G7 belonging to the same class (Table 2), again revealed pericyte-specific NG2/CSPG4 isoforms. However, the molecular species identified by these mAbs appeared more ubiquitous among the tumour vessels and through the pericyte strata. In fact, on double immunolabellings with Coll IV and VI, sites of colocalization coincided with the outer aspect of the Coll IV VBM rather than with Coll VI (Figure 5A-C), while in the innermost pericyte stratum 'niches' of proliferation were also seen, formed by 2164G7- and 2164G7/Coll VI-reactive pericytes (Figure S6A-C). In foetal brain, NG2/CSPG4 isoforms revealed by mAb 2161D7 appeared extensively distributed on the pericyte layer and also disclosed ramified cells. These NG2/CSPG4-reactive cells, never seen with other anti-NG2/CSPG4 clones, appeared sparse in the subventricular zone and more numerous in the intermediate zone (the emerging white matter in 22-week-old foetal telencephalon) (Figure 5D-F and Figure S7A-C), featuring a temporal and spatial distribution consistent with an OPCs (oligodendrocyte precursor cells) phenotype [45]. Some of them were identified by the O4-antigen, which recognizes specific glycolipids and cholesterol in OPCs in the course of their differentiation to pre-oligodendrocytes [46,47] (Figure S7D-F).

**Figure 6:**
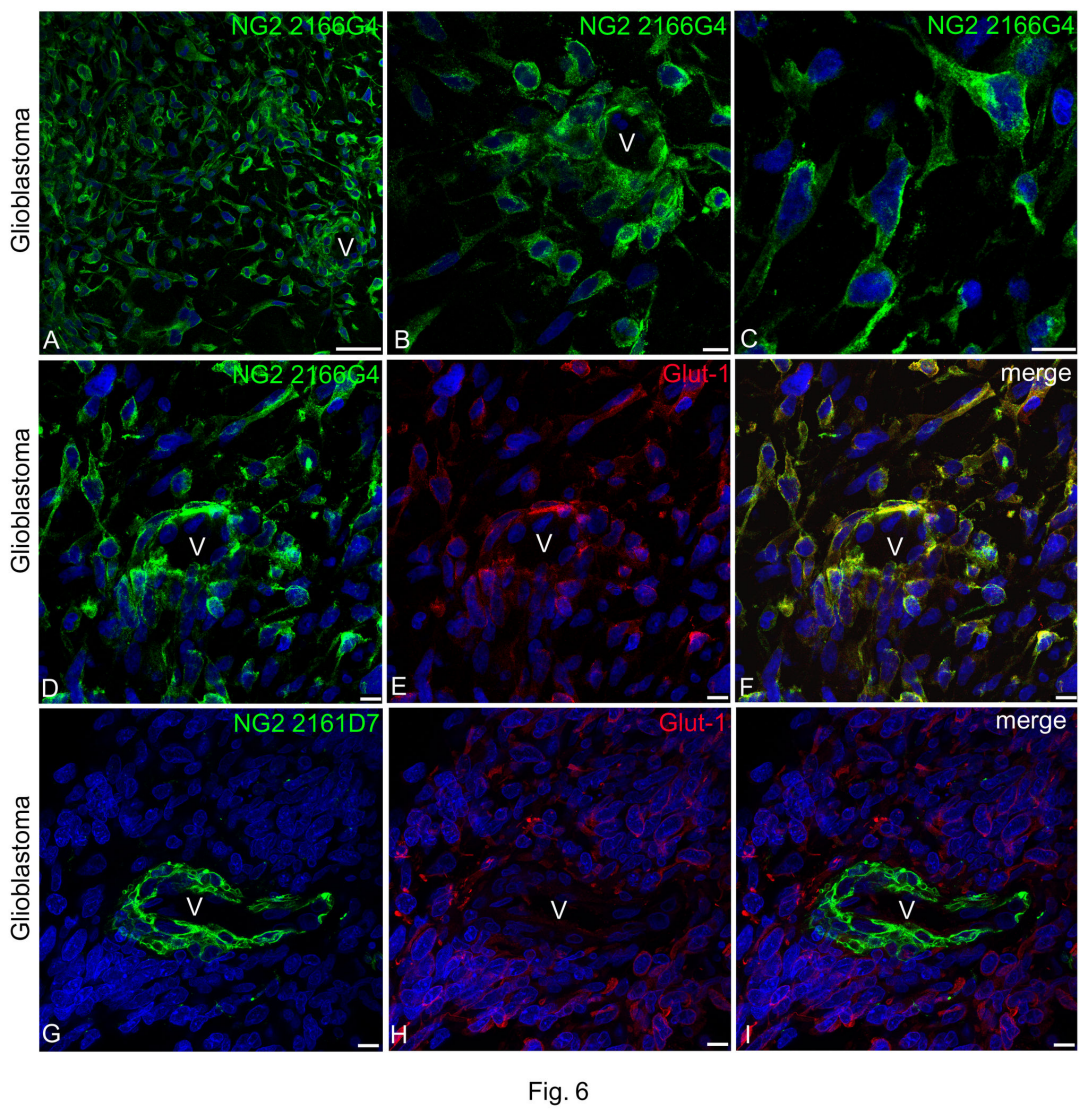
Expression of NG2/CSPG4 isoforms recognized by mAb 2166G4 in glioblastoma cells. (**A**-**C**) Immunolabelling with mAb 2166G4 shows the specific expression of these NG2/CSPG4 isoforms by the tumour cells, frequently seen forming perivascular cuffs (V in A and B). (**D**-**F**) Coincident staining of tumour cell 2166G4-reactive isoforms and Glut-1. (**G**-**I**) mAb 2161D7, identifying pericyte-specific NG2/CSPG4 isoforms, does not stain Glut-1-reactive tumoral cells that in this case do not infiltrate the vessel wall (V), providing indirect evidence of NG2/CSPG4 isoform specificity. Nuclear counterstaining TO-PRO3. Bars A 50 µm; B-I 10 µm.

The most unique NG2/CSPG4 isoform was found to be the one recognized by mAb 2166G4. This was noted to be exclusive of glioblastoma (Figure 6A-E), being entirely absent in foetal brain. In tumour tissues, mAb 2166G4 revealed tumour cells scattered in the stroma and forming typical perivascular cuffing, a distributional pattern that was never seen with the described pericyte-specific isoforms (i.e. with mAbs 2161F9 and 2161D7). The observation of a restricted expression of the isoform revealed by clone 2166G4 on neoplastic cells and its specificity as compared to the pericytes-specific ones, was verified by comparing 2166G4/Glut-1 and 2161D7/Glut-1 double immunolabellings (Figure 6D-F and G-I, respectively). Interestingly, Glut-1 was largely co-localized with the NG2/CSPG4 isoform detected by mAb 2166G4 on neoplastic cells scattered in the tissue and in the vicinity of tumour vessels (Figure 6D-F). On the contrary, Glut-1 was never seen to colocalize with the pericyte-specific isoform of the PG revealed by clone 2161D7, being detectable only on tumoral cells (Figure 6G-I).

### Immunochemical traits of NG2/CSPG4 isoforms expressed in foetal brain and glioblastoma

**Figure 7:**
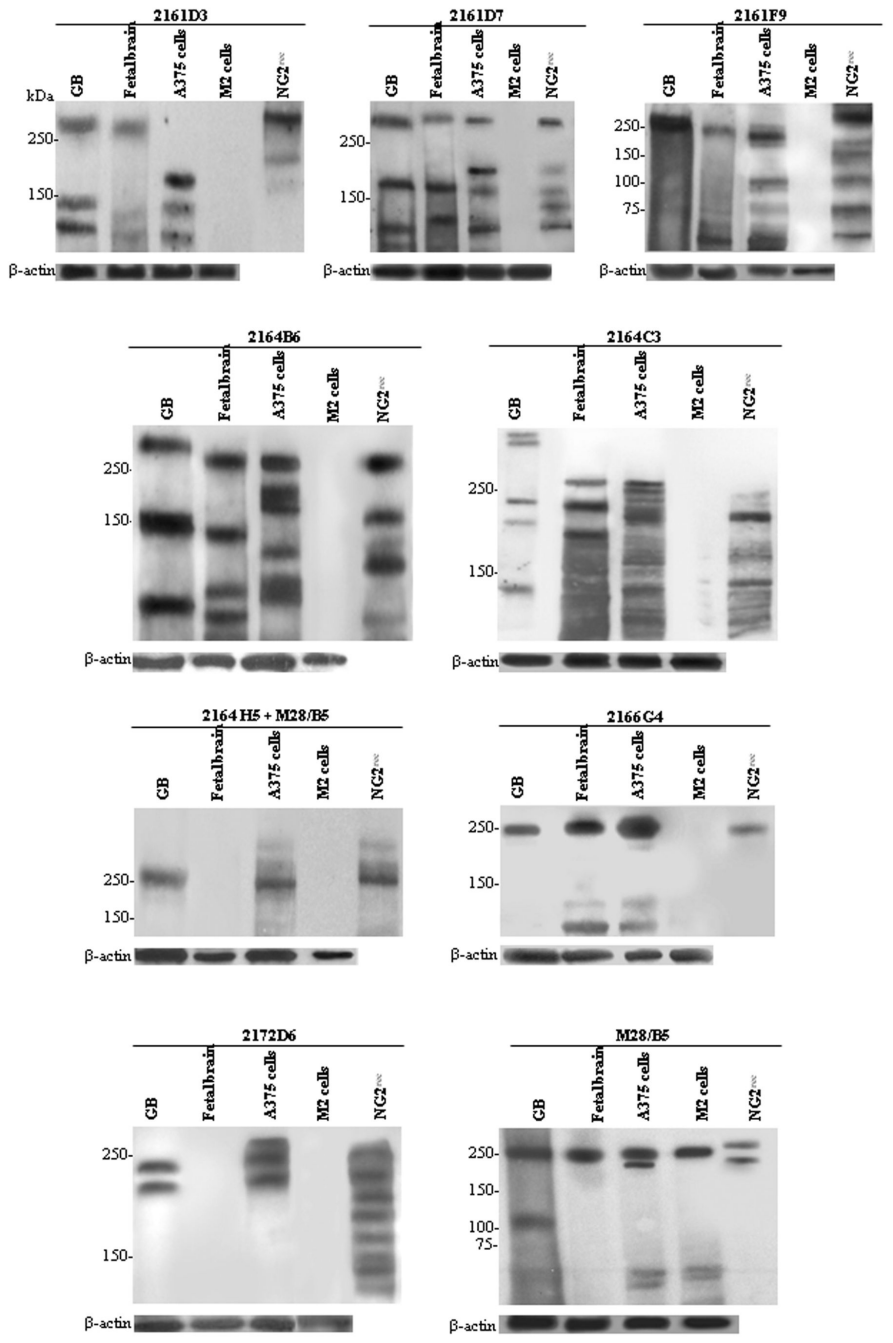
Immunochemical characteristics of NG2/CSPG4 isoforms and their proteolytic fragments in foetal brain, glioblastoma and neoplastic cells. Sections from foetal brain and glioblastoma samples were taken adjacently to sections in which abundant neovessels and enrichment of NG2/CSPG4-expressing pericytes were revealed by immunostaining. The sections were then processed for immunoblotting with the anti-NG2/CSPG4 mAbs (see M & M). A375 and M2 melanoma cells, and the recombinant NG2 ectodomain (NG2^rec^), used for producing the mAbs, were blotted in parallel for reference. Since mAb 2164H5 did not react with the cognate isoform in Western blotting, it was used to immunoprecipitate the isoform, which was then blotted with mAb B5/M28. The co-recognized isoform is likely to be only one of the NG2/CSPG4 variants and their cell surface-shed fragments immunoprecipitated by mAb 2164H5. Immunoblotting of α-actin with a polyclonal antiserum was used for normalization of gel lane loading. The relative position of molecular weight markers is indicated to the left of each panel.

Of the 30 mAbs recognizing the recombinant NG2 antigen in immunoblotting (NG2^rec^; Table 1), 8 reacted with NG2/CSPG4 isoforms expressed by foetal brain tissues or glioblastoma lesions, or both, yielding distinctive banding patterns (Figure 7).

In general, a more diversified banding pattern was observed with the recombinant NG2/CSPG4, suggesting that once expressed as an ectodomain fragment in HEK293 the PG was particularly susceptible to degradation by proteases produced by these cells. Primary proteolytic fragments of both foetal brain and glioblastoma ranged from 140-250 kDa. The melanoma cell line M2, although expressing high levels of NG2/CSPG4 in up to 90% of the cells (as determined by flow cytometry), failed to be recognized by any of the mAbs (Figure 7). The most striking difference was noted when comparing the isoforms recognized by the different antibodies in foetal brain, A375 cells and glioblastoma. Differences were particularly accentuated when considering the apparently diverse glycosylation patterns of the recognized NG2/CSPG4 isoforms of pericytes/OPCs versus tumour cells, i.e. as in the case of mAbs 2166G4 and 2172D6 versus mAb 2161F9 (Figure 7). Another interesting finding was that the isoforms recognized by the majority of mAbs in A375 melanoma cells were consistently less glycosylated than those expressed by pericytes/OPCs and possibly glioblastoma cells. This observation precludes the possibility that the mAbs reacted with discrete glycosylation isoforms, which in that case should have been the same for each tissue specimen. Sharper bands observed with several of the antibodies suggested that they recognized the NG2/CSPG4 core protein also in its partially unfolded form (i.e., during intracellular processing).

## Discussion

To provide insights into the cellular and molecular architecture of the angiogenic microvasculature of glioblastoma lesions, and compare their arrangement with those of healthy foetal and adult vasculature of the human brain, we generated a large panel of mAbs against the extracellular portion of human NG2/CSPG4 – a widely recognized critical surface component of active pericytes. We then made use of these antibodies to perform high-resolution fluorescence microscopy in conjunction with immune-mapping based upon endothelial markers and antibodies directed against perivascular ECM components. This approach allowed us to corroborate the expression of NG2/CSPG4 in subpopulations of glioblastoma cells [15-17] and disclose, for the first time, precisely organized pericyte subsets associated with nascent vessels of both foetal brain and glioblastoma. Furthermore, we were able to unveil NG2/CSPG4 isoforms that were pericytes-elective, i.e. not expressed by glioblastoma cells, or their associated non-neoplastic cells, and isoforms that appeared to be specific to the cancer cells as they were neither present on tumour vessels nor in foetal vessel pericytes.

A first characterization of the specificity of the anti-NG2/CSPG4 mAbs was based upon binding studies with tumour cell lines and indicated that the antibodies recognized NG2/CSPG4 variants that may in part be generated through differential post-translational modifications. Conversely, differential binding properties of the antibodies did not seem to be connected with diverse glycanation profiles of the PG. A more thorough characterization of the structural-compositional traits of these NG2/CSPG4 variants is therefore currently being pursued through extended protein sequence analyses. Some of the antibodies also reacted effectively with epitopes retained within fragments of the NG2/CSPG4 ectodomain, which were presumably released by cell surface proteolysis under both physiological and pathological conditions. Direct comparisons with the reactivity traits of antibodies reported in the literature further highlight their unique specificity for discrete variants of the PG. The possibility that the mAbs cross-reacted with other antigens could largely be excluded on the basis of a number of facts and empirical observations. Firstly, the uniqueness of the primary structure of NG2/CSPG4 within the human genome argues against the presence of immunodominant amino acid sequence stretches shared by other human proteins. Secondly, immunochemical reactivities were consistently abrogated by preincubation of the antibodies with the recombinant NG2/CSPG4 ectodomain used as immunogen. Thirdly, no reaction was seen with the antibodies when these were screened against cell lines *not* expressing NG2/CSPG4. Finally, mass spectrometric analyses, performed within the framework of a different ongoing study on NG2/CSPG4 molecules immunoprecipitated from different tumour cell lines, do not disclose peptide sequences belonging to other proteins.

As a first step toward the mapping of pericytes in glioblastoma vessels, we carried out a detailed analysis of the cellular arrangement characterizing the aberrant vascular structures known to be elaborated inside glioblastoma lesions, i.e. dilated, multilaminar “garland vessels" [10,36], and compared these structures to the angiogenic vessels of the developing human brain. Subsequent analyses based on the prototype mAbs 2161F9 and 2161D7 and other clones belonging to the same groups showed that both the developing brain and glioblastoma lesions embodied vessels made up of pericytes expressing more than one molecular form of NG2/CSPG4, and thereby discerned as discrete subsets.

In glioblastoma vessels, NG2/CSPG4-expressing pericytes were found to be associated with the VBM and to contribute to the 'garland' appearance of the vessels, identifying at least two phenotypically distinct and spatially separated pericyte populations. The internal one seemed to correspond to a population actively engaged in the construction of the vessel and possible extensions; to this subset also belonged the luminally arranged, dividing pericytes revealed. The other, more external population was often surrounded by an unusually stratified VBM that, at least in part, may have been deposited by the pericytes themselves. VBM multiple layers have been demonstrated by electron microscopy in tumour models of mouse carcinomas, where they are associated with structural abnormalities and may reflect tumour dynamics of endothelial cells and pericytes [43]. These two pericyte subsets were also polarized with respect to the luminal-abluminal axis of the vessel and accumulated surface molecules known to be critically involved in their vessel stabilizing function, including NG2/CSPG4 itself, PDGFRβ and endosialin/CD248. The precise functional properties of these subsets and how they are generated and maintained remain, of course, to be established. These observations provide concrete evidence at the single cell level of a spatio-functional distribution of tumour pericytes, which is directed toward the overlaying endothelium and may be instrumental in the pivotal interactions between the two cell types.

Apart from the typical pericyte-specific staining described with mAbs 2161F9 and 2161D7 and their relative clones, these two mAbs showed a significantly different potential for unveiling additional cell lineages. (i) In glioblastoma, mAb 2161F9 revealed pericyte precursor cells (PPCs) within the lumen of the tumour vessel network. These cells, which showed a progenitor-like small size and morphology with a high nucleus/cytoplasm ratio, lacked other markers of leukocytes and endothelial progenitor cells, such as CD3, CD31, CD45, CD146 and CD105, but expressed PDGFRβ that, in our double staining, was co-localized to a small degree with isoforms of NG2/CSPG4 revealed by mAb 2161F9, thus confirming that these NG2/CSPG4-reactive cells show, in part, a pericytic phenotype [44]. An increased frequency of PPCs was found in patients and mice with malignant tumours and in type 2 diabetic patients shown to have microangiopathy [23,44,48]. To the best of our knowledge, PPCs have never been described in glioblastoma; therefore, although an investigation of the role of PPCs is well beyond the scope of this work, we must not ignore the fact that these cells have been suggested to be involved in neoplastic perivascular cell differentiation, vascular survival, and cancer-related angiogenesis, aspects of primary importance in these extremely vascularized tumours [24]. (ii) In glioblastoma, mAb 2161D7 and other members of the same group demarcated NG2/CSPG4 isoforms that also reveal perivascular proliferating pericytes. Niches of proliferating pericytes are likely to be connected with tumour neovascularization phenomena, too. Early participation of pericytes in neovascularization has been described, shown by the formation of tubes (vasculogenic mimicry) and initiation of vascular sprouting [22,34,49,50]. Likewise, human bone marrow-derived mesenchymal stem cells (hMSCs) and glioblastoma stem cells (GSCs), pericytes of 'proliferative niches' might contribute to alternative mechanisms for the extension of the tumour vascular network by providing a reservoir of multipotent vascular progenitors [51-53]. (iii) In addition, in the developing brain, clone 2161D7 disclosed human OPCs (oligodendrocyte precursor cells), glial progenitor cells characterized by a NG2/O4-reactive phenotype that differentiate into myelinating oligodendrocytes and form a population of proliferative cells capable, in adult brain, of regenerating myelin following injury and disease [54,55].

Several of our mAbs belonging to the mAb 2164H5 group also allowed us to immunolocalize cell surface-shed NG2/CSPG4 fragments 'in situ' in both glioblastoma and foetal brain VBM and stromal ECM. In tumour vessels, NG2/CSPG4 fragments co-localized with Coll IV at the VBM/pericyte interface, forming clearcut rims denoted as 'pericyte lacunae', restricted specific domains pertaining to shed fragments and never seen to be co-localized with pericyte-specific isoforms identified by mAb 2161F9. Moreover, testifying to the origin of NG2/CSPG4 fragments not only from pericytes within the VBM but also from glioblastoma cells in the tumour ECM, mAb 2164H5 staining was present in proliferating tumour cells, that seemed to symmetrically segregate 2164H5-reactive NG2/CSPG4 isoforms into daughter cell cytoplasmic compartments [30]. Considering the involvement of the Golgi apparatus in post-translational glycoprocessing and its dynamic during mitosis, these may consist of Golgi vesicular and tubular membranes that are also segregated between the two daughter cells and, in telophase, fuse to reform the Golgi complex [56,57]. The idea is that tumour cells may produce an NG2/CSPG4 form as a secretory molecule, which is detectable during the phase of mitosis segregation to be thereafter released into the tumour ECM. Further studies are needed to support and elucidate the possible meaning of the observed data.

In contrast to the above ECM and pericyte-specific antibodies, mAb 2166G4 identified NG2/CSPG4 isoforms that were electively expressed by glioblastoma cells dispersed within the lesion and in vascular cuffs. Tumour cells were identified by co-expression of the glucose transporter isoform 1 (Glut-1). In normal conditions Glut-1 is a specific marker of blood-brain barrier (BBB)-ECs and has been considered to be an excellent indicator of a normally functioning barrier, changes in its expression being related to alterations in tight junctions and in turn in endothelial barrier/fence activities [58,59]. Glut-1 also appears to be the predominant glucose transporter in many types of cancer cells, where its overexpression is associated with an increased tumour invasiveness and poor clinical prognosis [60-62]. In glioblastoma, the reduction in Glut-1 expression parallels BBB-EC tight junction alterations and is consistent with BBB opening in tumour vessels [59,63]. By using Glut-1 as a counter-marker of neoplastic cells it was possible to visualize glioblastoma cells intermingled with pericytes around tumour vessels. The selectivity of the mAb 2166G4-reactive NG2/CSPG4 isoforms for neoplastic cells was also confirmed by the lack of expression of this isoform in both foetal and adult brain.

Comparative immunoblotting of tissue extracts derived from regions of the human foetal brain rich in NG2/CSPG4-expressing neovascular pericytes substantiated the presence of diverse isoforms of the PG on these cells and highlighted a widespread proteolytic fragmentation of the extracellular domain, which seemed different in human foetal brain as compared to glioblastoma lesions and cultured melanoma cell lines. This finding may be unexpected considering that, although tumours produce excessive amounts of metalloproteinase and other proteases capable of degrading NG2/CSPG4, such enzymes also accumulate in sprouting vessels [34]. The key difference explaining the discrepancy may be that a release of ECM-degrading enzymes during normal vessel sprouting is a temporally and locally controlled phenomenon, in direct contrast with the continuous ECM breakdown that ensues during tumour growth and invasion. Notably, angiogenic NG2/CSPG4 isoforms of foetal brain appeared overall to be more glycosylated than their cancer-associated isoform counterparts. The nature of this glycosylation and its biological significance remain to be defined. It remains similarly to be determined what may be the precise molecular identity of the NG2/CSPG4 fragments generated by sprouting vessels and whether release of these fragments contributes to the sequestering of extracellular signalling molecules (primarily growth factors), as previously shown for cell surface-shed syndecan fragments [64,65].

Overall, our findings provide the first demonstration of NG2/CSPG4 isoforms capable of recognizing healthy and neoplastic pericytes, and isoforms of the PG that effectively distinguish glioblastoma cells from discrete pericyte subsets. Post-translational modifications of cell membrane-associated molecules, truncated membrane-bound or soluble fragments resulting from proteolytic cleavage, as well as the suggested presence of tumour cell-derived secreted PG NG2/CSPG4 molecules, may all serve different biological activities whose roles in glioblastoma vascularization and growth have still to be ascertained. The observations also highlight clearly delineated arrangements of perivascular cells whose precise functional implications deserve further attention. A precise identification of molecules selectively expressed by the tumour vasculature and associated with vascular sprouting will contribute to a better understanding of structural-functional characteristics of the vascular microenvironment in healthy and diseased CNS and may have a significant impact on the development of combined anti-neoplastic/anti-angiogenic therapeutic approaches.

## Supporting Information

Figure S1 **Absence of NG2/CSPG4-expressing pericytes in microvessels of adult normal brain.** Human parahippocampal cortex was double-stained with an antibody to Coll IV and the pAb NG2 D2 recognizing pericyte-specific NG2/CSPG4 isoforms and their proteolysis products (**A**, **B**), or mAbs directed against various isoforms of the PG (**C**-**F**). The Coll IV-containing VBMs are characteristically seen surrounding the entire pericyte body that appears unstained (arrows). Nuclear counterstaining TO-PRO3. Bars 10 µm.
(TIF)

Figure S2 **Prototype mAbs as emerged from data summarized in Table 2 and their representative staining pattern obtained on paraffin embedded glioblastoma lesions.** mAb 2164H5 recognizes fragments of NG2/CSPG4 isoforms released from the cell surface into the vascular basement membrane (VBM) and extracellular matrix (ECM). mAbs 2161F9 and 2161D7 identify pericyte isoforms of the proteoglycan and mAb 2166G4 identify NG2/CSPG4 isoforms expressed by tumour cells. Semi-quantitative scoring of immunoreactivity: “–“, very weak or absent; “+”, weak; “++”, intermediate ; “+++”, strong; “++++”, very strong.
(TIF)

Figure S3 **Representative images of vascular patterning in different glioblastoma areas.** (**A**) Most glioblastoma peripheral regions show a rich network of small neoformed vessels revealed by Coll IV; (**B**) small vessels characterized by an NG2 D2-reactive thick wall occupy central areas of the tumour tissue; (**C**) a typical 'garland' vessel revealed by NG2 D2, that also comprises capillary tufts (*asterisks*). *Bars* 25 µm.
(TIF)

Figure S4 **Subcellular localization of pericyte markers, PDGFRβ and αSMA, in double stainings with NG2 D2 on glioblastoma vessels.** (**A**-**C**) The antibody against the phosphorylated form of PDGFRβ selectively identifies the activated receptor on the pericyte adluminal front. (**D**-**F**) Clear-cut images of the differential localization of αSMA and NG2 D2 in pericytes, even better recognizable in the region of the nucleus (enlarged in the inset), actin appears concentrated in the sublemmal cell compartment (*arrow*), whereas NG2 D2 is distributed on the luminal plasma membrane (*arrowhead*). Nuclear counterstaining TO-PRO3. *Bars* A-C 25 µm; D-F 10µm.
(TIF)

Figure S5 **PPCs revealed by mAb 2161F9 co-express the pericyte marker PDGFRβ.** (**A**-**C**) The tumour vessel, double-labelled by pAb NG2 D2 and mAb 2161F9, shows an intraluminal pericyte precursor-like 2161F9-stained cell and its possible passageway across the tumour vessel endothelium (*arrow*); in the corresponding enlargement (**A**'-**C**'), confocal laser detection was maximized to reveal the endothelial cell profile thanks to the autofluorescence of these cells in both the laser channels. (**D**-**E**) By double staining with the pericyte marker PDGFRβ, the pericyte nature of an NG2 2161F9-reactive cell is confirmed (*arrowhead*). Nuclear counterstaining TO-PRO3. *Bars* A-C 20 µm; A'-C' 5 µm.
(TIF)

Figure S6 **Proliferating pericytes disclosed in glioblastoma vessels by expression of specific NG2/CSPG4 isoforms.** (**A**-**C**) Double staining with anti-Coll VI and mAb 2164G7 discloses actively dividing pericytes in different phases of mitosis; high magnification views in **A**'**-C**'; note in **C'** the dividing pericyte that also stains for Coll VI. Nuclear counterstaining TO-PRO3. *Bars* A-C 10 µm; A'-C' 5 µm.
(TIF)

Figure S7 **Foetal brain OPCs immunolabelled by NG2 2161D7 and O4.** (**A**-**C**) Single staining with mAb 2161D7 reveals a wave of migrating OPCs (*arrows*) in the intermediate zone of the telencephalic wall (**A**) and the typical ramified morphology of these precursor cells (**B**, **C**). (**D**-**F**) By double staining with the oligodendrocyte marker O4, the OPC nature of the NG2 2161D7-reactive cells is confirmed; note a differentiating OPC whose body only expresses the O4 marker (*arrowhead*). Nuclear counterstaining TO-PRO3. *Bars* A 25 µm; B-F 10 µm.
(TIF)

Movie S1 **Sequence of single optical plane from the z-stack image shown in Figure 1B; CD31/Collagen IV immunolabelling.** CD31-stained microvesicles appear released from the cell surface into the vessel lumen.
(AVI)

Movie S2 **Sequence of single optical plane from the z-stack image shown in Figure 1G; CD248/NG2D2 immunolabelling.** CD248- and NG2D2-expressing pericytes appears as two spatially separated populations within the tumour vessel wall.
(AVI)

Table S1 **Complete list of primary and secondary antibodies.**
(DOCX)
