# Correction: Diversified Expression of NG2/CSPG4 Isoforms in Glioblastoma and Human Foetal Brain Identifies Pericyte Subsets

**DOI:** 10.1371/journal.pone.0095120

**Published:** 2014-04-09

**Authors:** The PLOS ONE Staff

## Notice of Republication

This article was republished on March 14, 2014, to replace Tables 1 and 2, which were originally published incorrectly. The publisher apologizes for these errors. Please download this article again to view the correct version.
