# Supplementary figures and images for: Diversified Expression of NG2/CSPG4 Isoforms in Glioblastoma and Human Foetal Brain Identifies Pericyte Subsets
Source: PLoS One. 2013 Dec 26;8(12):e84883. doi: 10.1371/journal.pone.0084883 (PMC3873429; doi:10.1371/journal.pone.0084883)

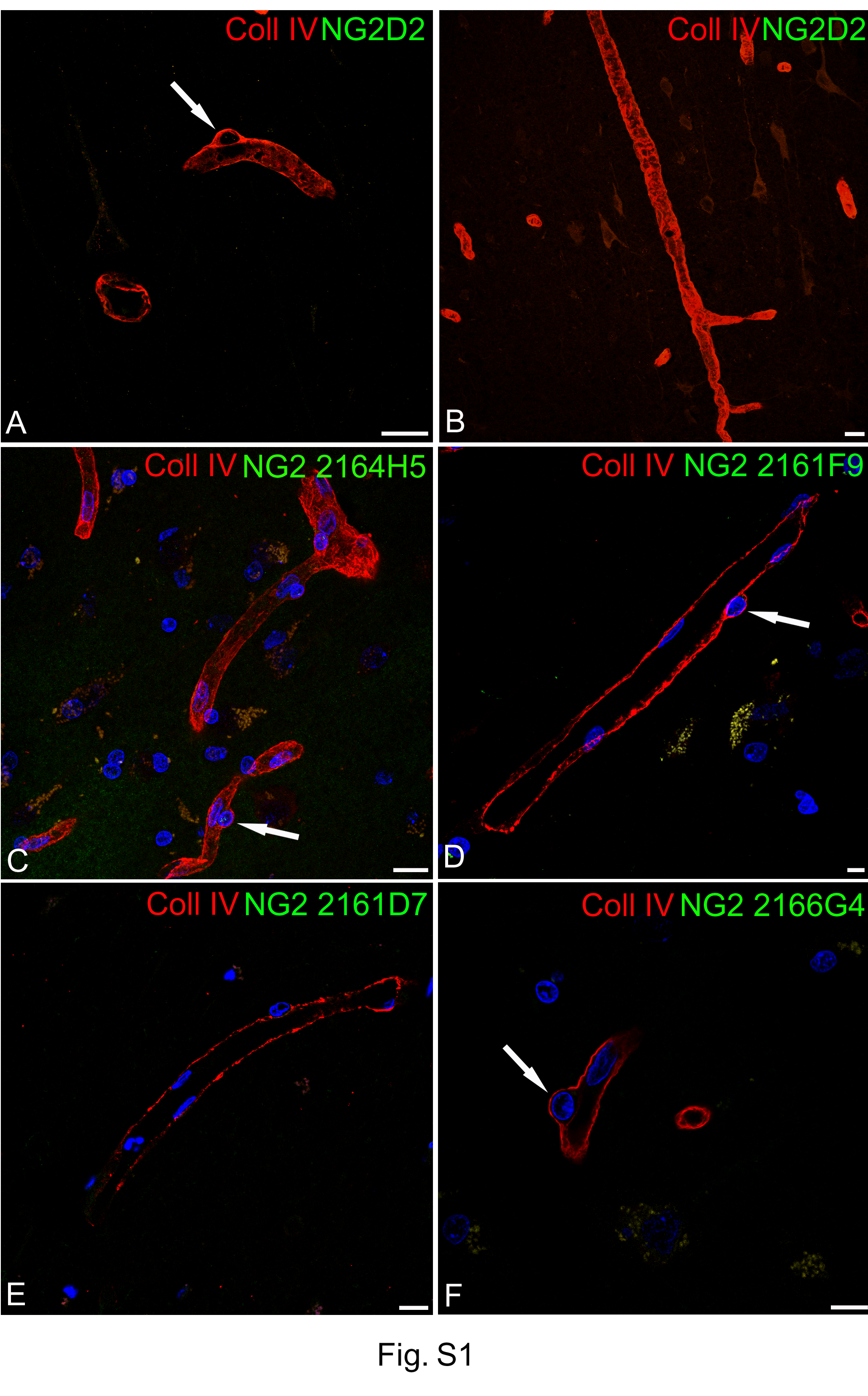

Supplement: Figure S1 — Absence of NG2/CSPG4-expressing pericytes in microvessels of adult normal brain. Human parahippocampal cortex was double-stained with an antibody to Coll IV and the pAb NG2 D2 recognizing pericyte-specific NG2/CSPG4 isoforms and their proteolysis products (A, B), or mAbs directed against various isoforms of the PG (C-F). The Coll IV-containing VBMs are characteristically seen surrounding the entire pericyte body that appears unstained (arrows). Nuclear counterstaining TO-PRO3. Bars 10 µm. (TIF) [file pone.0084883.s001.tif]

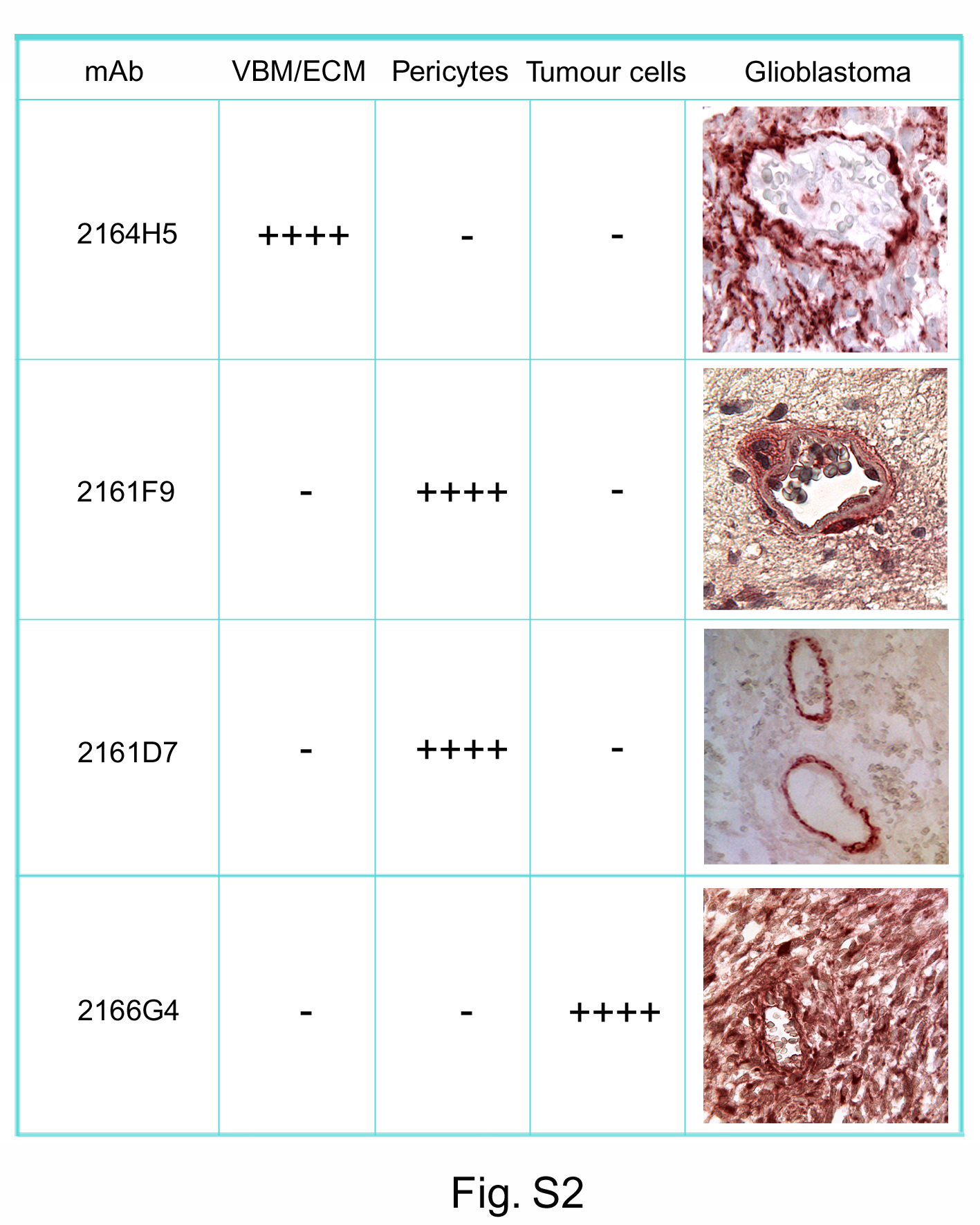

Supplement: Figure S2 — Prototype mAbs as emerged from data summarized in Table 2 and their representative staining pattern obtained on paraffin embedded glioblastoma lesions. mAb 2164H5 recognizes fragments of NG2/CSPG4 isoforms released from the cell surface into the vascular basement membrane (VBM) and extracellular matrix (ECM). mAbs 2161F9 and 2161D7 identify pericyte isoforms of the proteoglycan and mAb 2166G4 identify NG2/CSPG4 isoforms expressed by tumour cells. Semi-quantitative scoring of immunoreactivity: “–“, very weak or absent; “+”, weak; “++”, intermediate ; “+++”, strong; “++++”, very strong. (TIF) [file pone.0084883.s002.tif]

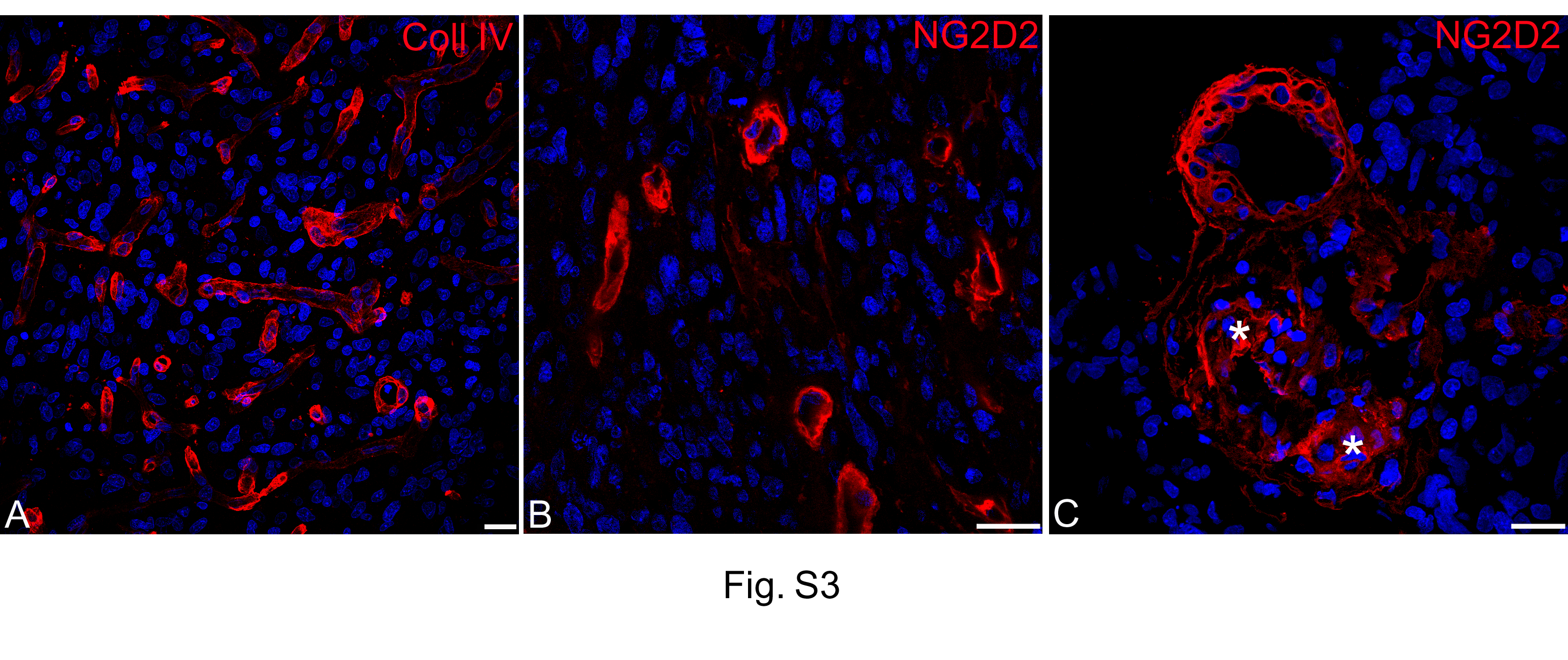

Supplement: Figure S3 — Representative images of vascular patterning in different glioblastoma areas. (A) Most glioblastoma peripheral regions show a rich network of small neoformed vessels revealed by Coll IV; (B) small vessels characterized by an NG2 D2-reactive thick wall occupy central areas of the tumour tissue; (C) a typical 'garland' vessel revealed by NG2 D2, that also comprises capillary tufts (asterisks). Bars 25 µm. (TIF) [file pone.0084883.s003.tif]

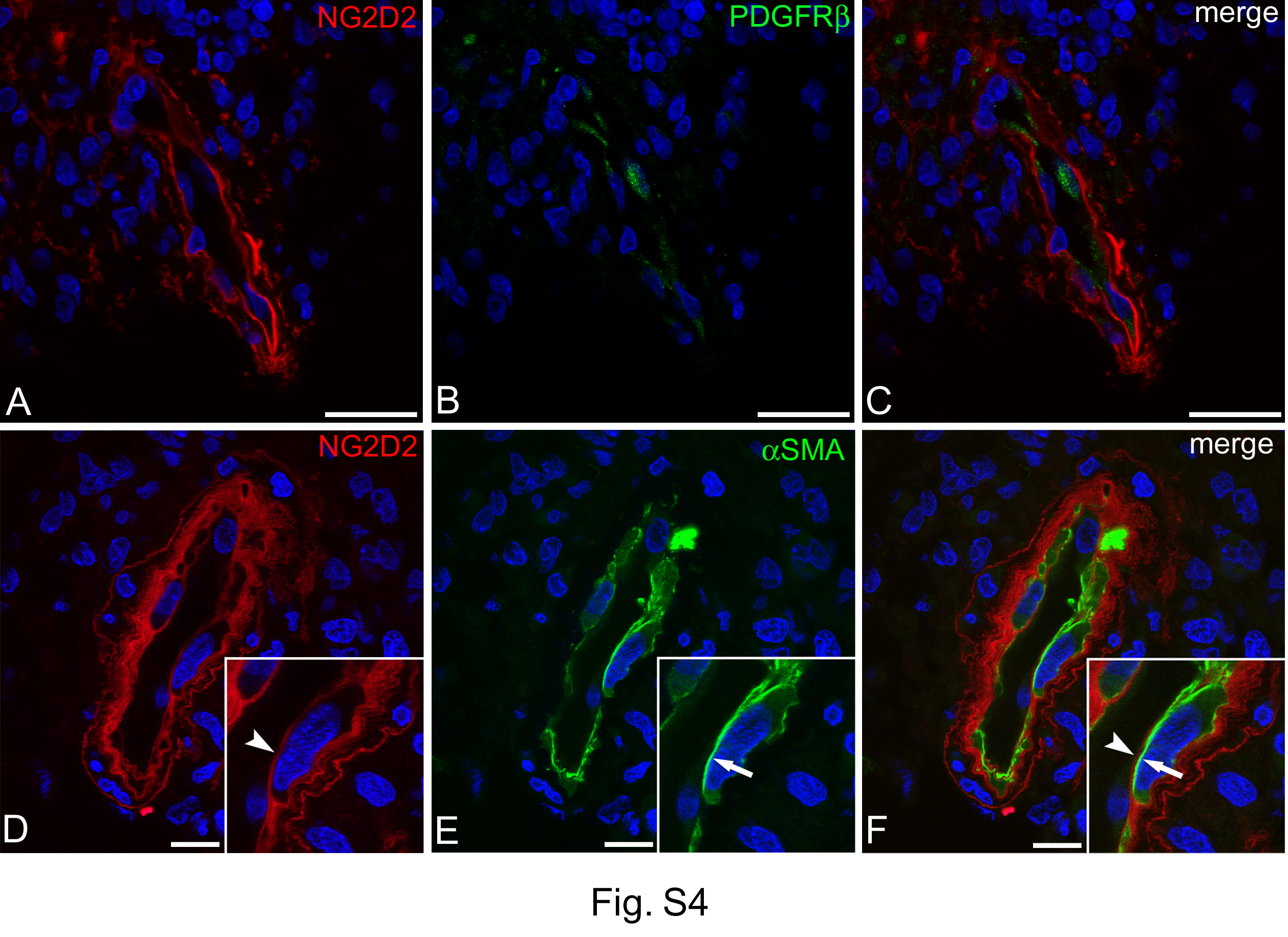

Supplement: Figure S4 — Subcellular localization of pericyte markers, PDGFRβ and αSMA, in double stainings with NG2 D2 on glioblastoma vessels. (A-C) The antibody against the phosphorylated form of PDGFRβ selectively identifies the activated receptor on the pericyte adluminal front. (D-F) Clear-cut images of the differential localization of αSMA and NG2 D2 in pericytes, even better recognizable in the region of the nucleus (enlarged in the inset), actin appears concentrated in the sublemmal cell compartment (arrow), whereas NG2 D2 is distributed on the luminal plasma membrane (arrowhead). Nuclear counterstaining TO-PRO3. Bars A-C 25 µm; D-F 10µm. (TIF) [file pone.0084883.s004.tif]

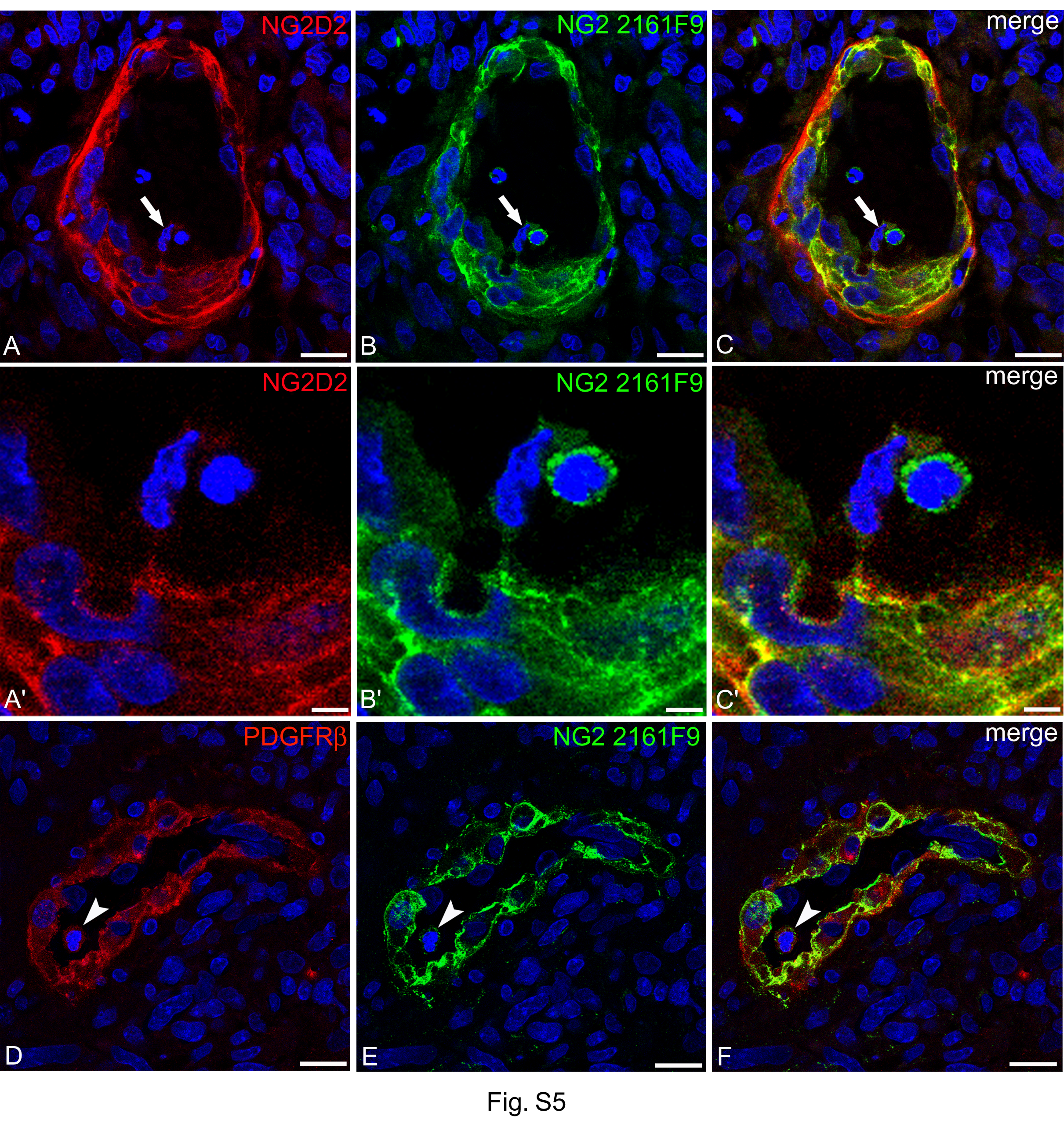

Supplement: Figure S5 — PPCs revealed by mAb 2161F9 co-express the pericyte marker PDGFRβ. (A-C) The tumour vessel, double-labelled by pAb NG2 D2 and mAb 2161F9, shows an intraluminal pericyte precursor-like 2161F9-stained cell and its possible passageway across the tumour vessel endothelium (arrow); in the corresponding enlargement (A'-C'), confocal laser detection was maximized to reveal the endothelial cell profile thanks to the autofluorescence of these cells in both the laser channels. (D-E) By double staining with the pericyte marker PDGFRβ, the pericyte nature of an NG2 2161F9-reactive cell is confirmed (arrowhead). Nuclear counterstaining TO-PRO3. Bars A-C 20 µm; A'-C' 5 µm. (TIF) [file pone.0084883.s005.tif]

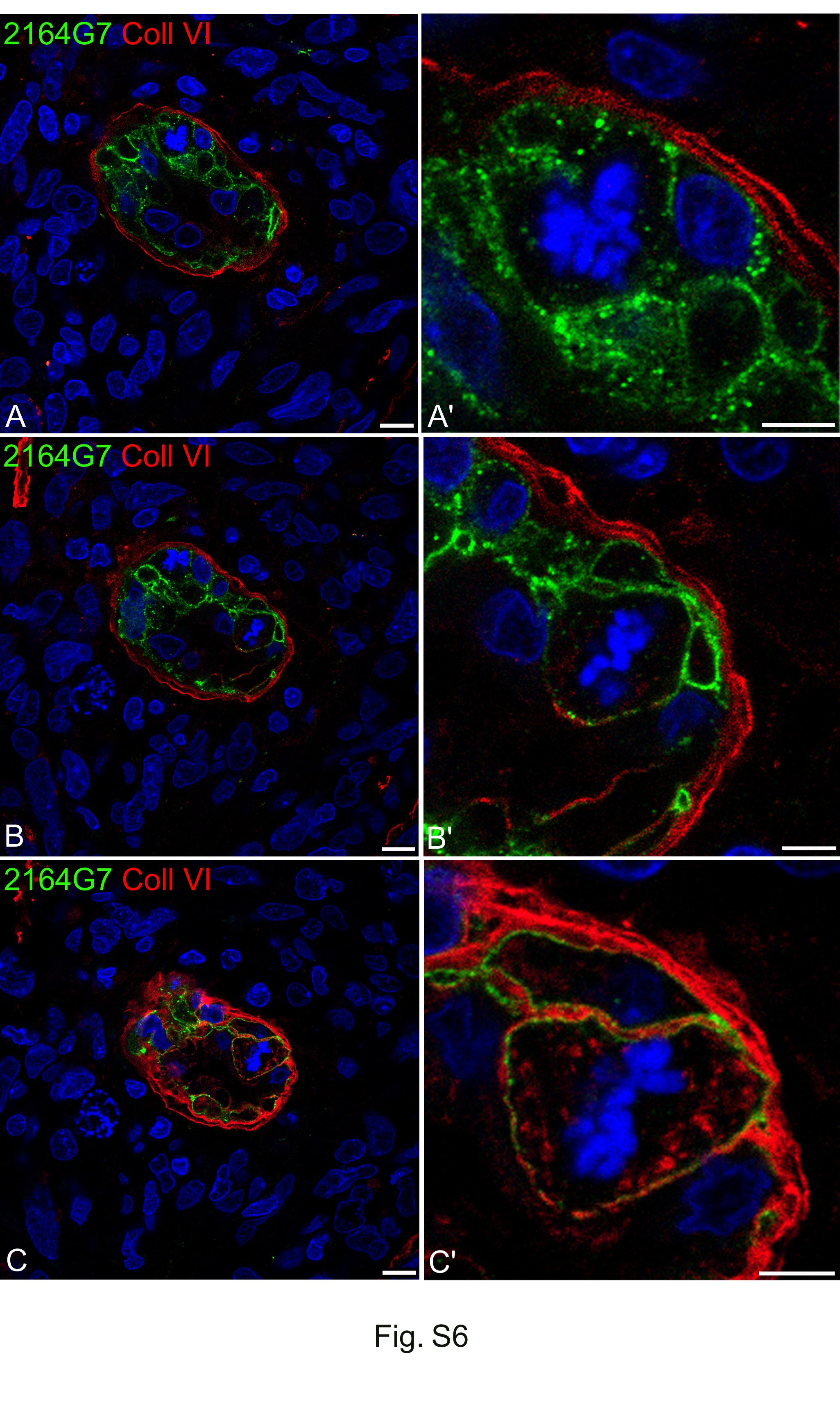

Supplement: Figure S6 — Proliferating pericytes disclosed in glioblastoma vessels by expression of specific NG2/CSPG4 isoforms. (A-C) Double staining with anti-Coll VI and mAb 2164G7 discloses actively dividing pericytes in different phases of mitosis; high magnification views in A'-C'; note in C' the dividing pericyte that also stains for Coll VI. Nuclear counterstaining TO-PRO3. Bars A-C 10 µm; A'-C' 5 µm. (TIF) [file pone.0084883.s006.tif]

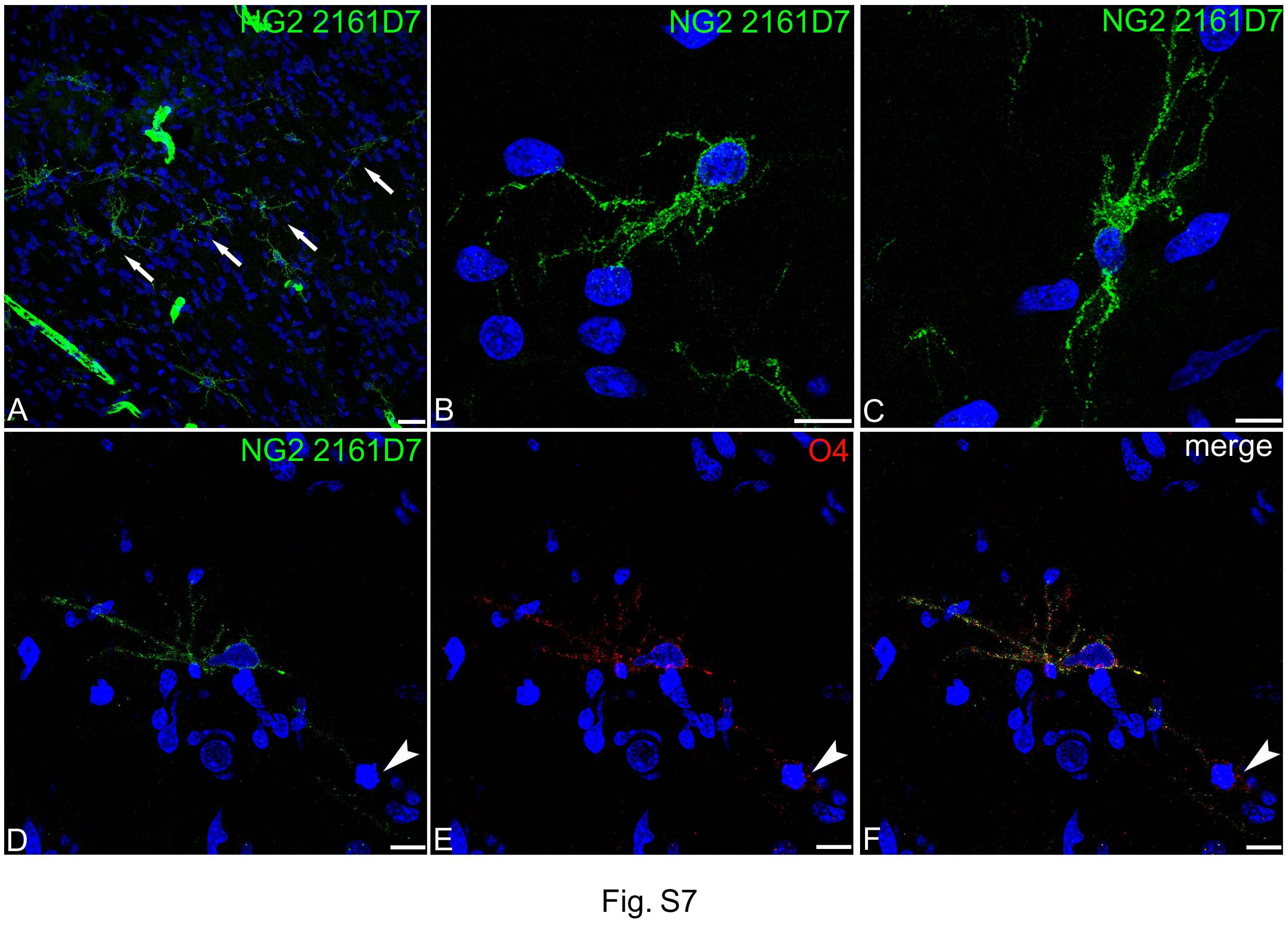

Supplement: Figure S7 — Foetal brain OPCs immunolabelled by NG2 2161D7 and O4. (A-C) Single staining with mAb 2161D7 reveals a wave of migrating OPCs (arrows) in the intermediate zone of the telencephalic wall (A) and the typical ramified morphology of these precursor cells (B, C). (D-F) By double staining with the oligodendrocyte marker O4, the OPC nature of the NG2 2161D7-reactive cells is confirmed; note a differentiating OPC whose body only expresses the O4 marker (arrowhead). Nuclear counterstaining TO-PRO3. Bars A 25 µm; B-F 10 µm. (TIF) [file pone.0084883.s007.tif]
