# Supplementary material for: Diversified Expression of NG2/CSPG4 Isoforms in Glioblastoma and Human Foetal Brain Identifies Pericyte Subsets
Source: PLoS One. 2013 Dec 26;8(12):e84883. doi: 10.1371/journal.pone.0084883 (PMC3873429; doi:10.1371/journal.pone.0084883)
Supplement: Table S1 — Complete list of primary and secondary antibodies. (DOCX) [file pone.0084883.s010.docx]

***Table S1.*** *Complete list of primary and secondary antibodies*

| Primary antibodies | Host IgG | Dilution | | Producer^1^ | Code number | |  |
| --- | --- | --- | --- | --- | --- | --- | --- |
| α-SMA | mouse IgG_2a,k_ | | 1:30 | Dako | | N 1584 | |
| CD3 | rabbit IgG | | 1:50 | Abcam | | AB5690 | |
| CD31 | mouse IgG_1,k_ | | 1:10 | Dako | | M 0823 | |
| CD45 | rat IgG_2_ | | 1:50 | Novus Biol. | | NB110-93609 | |
| CD105 | rabbit IgG | | prediluted | Millipore | | MAB5384 | |
| CD146 | mouseIgG_1_ | | 1:50 | Abcam | | AB49492 | |
| CD248 | mouse IgG | | 1:250 | C.M. Isacke | | - | |
| Coll IV | rabbit IgG | | 1:50 | Acris | | R1041 | |
| Coll IV | mouse IgG | | 1:100 | Millipore | | AB5320 | |
| Coll VI | rabbit IgG | | 1:100 | Abcam | | AB6588 | |
| Glut 1 | rabbit IgG | | 1:100 | Millipore | | 071401 | |
| MMP-2 | mouse IgG_1_ | | 1:50 | Millipore | | MAB13431 | |
| NG2D2 | rabbit IgG | | 1:50 | W.B. Stallcup | | - | |
| O4 | mouse IgM | | 1:10 | Millipore | | MAB345 | |
| PDGFR-β | goat IgG | | 1:50 | R&D Systems | | AF385 | |
| Phospho-PDGFR-β | mouse IgG_2b_ | | 1:50 | Cell Signaling | | 3166 | |
| Secondary antibodies | | Dilution | | Producer | Code number | |  |
| biotinylated goat anti-rabbit | | | 1:400 | Vector | | BA-1000 | |
| biotinylated horse anti-mouse | | | 1:400 | Vector | | BA-2000 | |
| biotinylated goat anti-rat | | | 1:300 | Vector | | BA-9400 | |
| biotinylated goat anti mouse IgM | | | 1:300 | Invitrogen | | D20693 | |
| streptavidin-conjugated Alexa 488 | | | 1:400 | Invitrogen | | S-32354 | |
| streptavidin-conjugated Alexa 555 | | | 1:300 | Invitrogen | | S-32355 | |
| goat anti-rabbit Alexa 568 | | | 1:400 | Invitrogen | | A11011 | |
| goat anti-mouse Alexa 555 | | | 1:400 | Invitrogen | | A21425 | |
| donkey anti-goat Alexa 633 | | | 1:300 | Invitrogen | | A21082 | |

^1^Abcam, Cambridge, UK; Dako Cytomation, Glostrup, Denmark; Novus Biological, Littleton, CO, USA; Millipore-Chemicon; Billerica, MA, USA; Acris Antibodies GmbH; Herford, Germany; Cell Signalling Technology; Beverly, MA, USA; Vector Laboratories Inc., Burlingame, CA, USA; Invitrogen, Eugene, OR, USA; R&D Systems, Minneapolis, MN, USA;
